# Contribution of specific ceramides to obesity-associated metabolic diseases

**DOI:** 10.1007/s00018-022-04401-3

**Published:** 2022-07-05

**Authors:** Philipp Hammerschmidt, Jens C. Brüning

**Affiliations:** 1 Department of Neuronal Control of Metabolism, Max Planck Institute for Metabolism Research, Gleueler Strasse 50, 50931 Cologne, Germany; 2 Center for Endocrinology, Diabetes and Preventive Medicine (CEDP), University Hospital Cologne, Kerpener Strasse 26, 50924 Cologne, Germany; 3 Cologne Excellence Cluster on Cellular Stress Responses in Aging-Associated Diseases (CECAD) and Center for Molecular Medicine Cologne (CMMC), University of Cologne, Cologne, Germany; 4 National Center for Diabetes Research (DZD), Ingolstädter Landstrasse 1, 85764 Neuherberg, Germany

**Keywords:** Atherosclerosis, Ceramide acyl chain length, Sphingolipids, Lipid signaling, Lipotoxicity, High-fat diet, Obesity, Insulin resistance, Diabetes, Metabolic disease treatment

## Abstract

Ceramides are a heterogeneous group of bioactive membrane sphingolipids that play specialized regulatory roles in cellular metabolism depending on their characteristic fatty acyl chain lengths and subcellular distribution. As obesity progresses, certain ceramide molecular species accumulate in metabolic tissues and cause cell-type-specific lipotoxic reactions that disrupt metabolic homeostasis and lead to the development of cardiometabolic diseases. Several mechanisms for ceramide action have been inferred from studies in vitro, but only recently have we begun to better understand the acyl chain length specificity of ceramide-mediated signaling in the context of physiology and disease in vivo. New discoveries show that specific ceramides affect various metabolic pathways and that global or tissue-specific reduction in selected ceramide pools in obese rodents is sufficient to improve metabolic health. Here, we review the tissue-specific regulation and functions of ceramides in obesity, thus highlighting the emerging concept of selectively inhibiting production or action of ceramides with specific acyl chain lengths as novel therapeutic strategies to ameliorate obesity-associated diseases.

## Background

Obesity rates have increased alarmingly over the past 50 years among both adults and children [1, 2], urging the WHO to describe obesity as “one of today’s most blatantly visible—yet most neglected—public health problems” [3]. Obesity is a complex, multifactorial disease of excess adiposity that can cause premature disability and death by increasing the risk of metabolic disorders such as type 2 diabetes mellitus, fatty liver disease, and cardiovascular impairment, mainly due to dyslipidemia and ectopic lipid deposition [4]. However, efforts to prevent or treat obesity and its comorbidities often fail in the long term, and available pharmacotherapeutics remain primarily ineffective and unspecific [5]. Thus, there is an urgent clinical need to better understand the physiological and molecular mechanisms linking obesity to metabolic deterioration in order to identify novel targets for future therapeutic interventions.

Many people with obesity show elevated levels of plasma free fatty acids (FFAs), which is partly attributable to unopposed lipolysis in adipocytes secondary to decreased insulin sensitivity and impaired adipose tissue function [6]. As a result, FFAs target organs such as the liver, muscle, pancreas, heart, and central nervous system, where they can be utilized, stored ectopically in an inert storage pool as triacylglycerols (TAG), or used for the production of other lipid species involved in regulating various metabolic processes [7]. However, when the maximal capacity for fatty acid oxidation or TAG deposition is reached, specific lipid metabolites accumulate that can cause cell-type-specific adverse reactions (referred to as lipotoxicity) and promote metabolic dysfunction such as local and systemic insulin resistance with far-reaching health consequences [7, 8].

In the early 1990s, it was found that specific sphingolipids, namely ceramides, accumulate in the liver and muscle of obese and diabetic rats [9]. Ceramides are composed of a sphingoid long-chain base attached to a single fatty acid. Later, ceramide levels in body tissue and plasma were correlated with diminished insulin sensitivity among obese and type 2 diabetic patients [10, 11]; and in 2007, increased endogenous ceramide synthesis came into the spotlight as to cause insulin resistance in vivo [12]. Meanwhile, it has been found that ceramides accrue in many other metabolic tissues in obesity, and numerous lipotoxic responses were attributed to ceramide action. Ceramides modulate cell membrane dynamics, endoplasmic reticulum (ER) and mitochondrial integrity, inflammation, and cell fate [13]. Notably, ceramides form a family of closely related but structurally and functionally diverse molecular species that differ in sphingoid base composition as well as length and saturation of the acyl chain [14, 15]. Depending on the acyl chain length, most commonly ranging from 14 to 26 carbons (C_14_-C_26_), ceramides produce distinct pathophysiological effects and accumulate differentially within each cell type and cell compartment, while causing a range of adverse consequences associated with obesity [16]. As a result, reducing selected pools of ceramides has proven sufficient for preventing the detrimental effects of fatty acid excess and—even more excitingly—for ameliorating metabolic homeostasis in obese murine models, with a significantly lower risk of adverse side effects than would ensue from the complete inhibition of global ceramide formation [16].

This review discusses the emerging concept of the ceramide species-specific regulation of metabolism in obesity, focusing on “classic ceramide species” that contain the typical sphingoid base sphingosine (d18:1) and a saturated acyl chain of defined length. We present the basic principles of mammalian ceramide turnover and highlight key aspects of their pathophysiological roles. We discuss a selection of pathways that employ ceramides as second messengers by controlling ceramide metabolic rate and thus contribute to ceramide accumulation when deregulated in obesity. Finally, we outline the tissue-specific regulation of ceramides in obesity and how this knowledge could be translated into clinics for the treatment of metabolic diseases. Thereby, we aim to provide an updated view of “the complex life of (these) simple sphingolipids”—as Hannun and Futerman once put it [17]—in the context of physiology, lipotoxicity, obesity-associated pathologies, and their treatment.

## Metabolism of ceramides in mammals

**Fig. 1:**
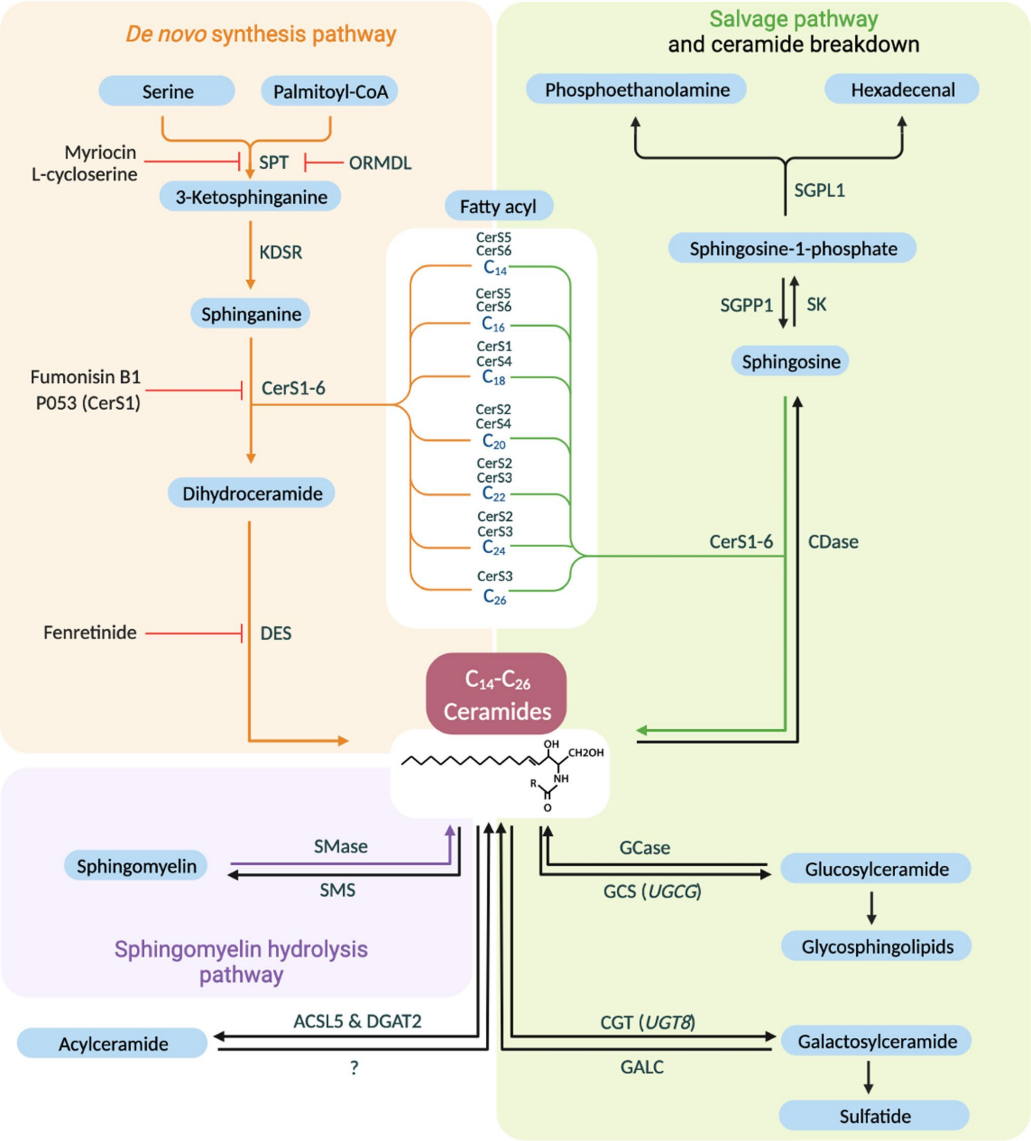
Ceramide metabolism in mammals. Schematic representation of the ceramide metabolic pathway highlighting critical enzymes involved in ceramide turnover and their respective inhibitors. Six different ceramide synthases (CerS1-6) produce (dihydro)ceramides of varying acyl chain lengths by catalyzing the N-acylation of sphinganine (derived from the condensation of serine and palmitoyl-CoA; de novo pathway; highlighted in orange) or sphingosine (derived from sphingolipid breakdown; salvage pathway; highlighted in green) with a fatty acyl chain of defined length within the range C_14_–C_26_. Ceramides can also be derived from the hydrolysis of sphingomyelin (highlighted in purple). Ceramides serve as substrates for more complex sphingolipid species such as glucosylceramides and galactosylceramides, which can be further modified. Ceramides can also be converted to acylceramide species bearing an additional acyl chain at the 1-hydroxy position. *ACSL5* Acyl-CoA synthetase long-chain family member 5, *CDase* ceramidase, *CerS* ceramide synthase, *CGT *ceramide UDP-galactosyltransferase, *DEGS* dihydroceramide desaturase, *DGAT2* diacylglycerol *O*-acyltransferase 2, *GALC* galactosylceramidase, *GCase* glucocerebrosidase, *GCS* glucosylceramide synthase, *KDSR* 3-ketodihydrosphingosine reductase, *ORMDL* orosomucoid-like protein, *R* Fatty acyl chain moiety, *SGPL1* sphingoine-1-phosphate lyase 1, *SGPP1* sphingosine-1-phosphate phosphatase 1, *SK* sphingosine kinase, *SMase* sphingomyelinase, *SMS* sphingomyelin synthase, *SPT* serine palmitoyltransferase, *UGCG* UDP-glucose ceramide glucosyltransferase, *UGT8* UDP glycosyltransferase

Ceramides are a family of ubiquitous, bioactive lipid molecules that serve as the structural unit of all more complex sphingolipid species. These comprise a set of aliphatic amino alcohols with a backbone of sphingoid long-chain bases. Ceramides consist of the sphingoid scaffold bound to a fatty acid via amide-linkage, and they vary in length and degree of unsaturation within both aliphatic components depending on the biological origin [18]. Three separate routes of endogenous ceramide formation can be distinguished, i.e., de novo synthesis, sphingomyelin hydrolysis, and sphingolipid salvage (Fig. 1). The canonical de novo synthesis pathway commences with the production of the long-chain base by condensation of serine and palmitoyl-CoA to form 3-ketosphinganine at the cytosolic surface of the ER. This reaction is catalyzed by serine palmitoyltransferase (SPT), an enzyme complex composed of two ubiquitously expressed large subunits, encoded by *Sptlc1* and -*2*, and a small regulatory subunit [19]. *Sptlc3* encodes an alternative large subunit forming a spectrum of straight and branched long-chain bases with distinct biophysico-chemical properties in restricted tissues [20]. The carbonyl group of 3-ketosphinganine is subsequently reduced by 3-ketodihydrosphingosine reductase (KDSR) to form sphinganine, which can become acylated with a fatty acyl-CoA of defined length (C_14_-C_26_) by one of six (dihydro)ceramide synthases (CerS1-6; see Box 1) to form dihydroceramide [21]. Ultimately, two distinct dihydroceramide desaturases (DES1 and DES2, encoded by *Degs1* and *Degs2*) integrate a 4,5-*trans*-double bond into the sphingoid base to produce ceramides, with DES1 responsible for ceramide synthesis in most tissues [22]. Each ceramide species appears to contain a fixed acyl chain length, as there is currently no evidence for remodeling after ceramide formation. Studies in rodent models have indicated that the expression of ceramide biosynthetic genes increase in obesity and that interventions to reduce ceramide synthesis either by genetic modification (e.g., ablation of *Sptlc2* [23, 24], *Degs1* [12, 25], *CerSs* [26–28]) or pharmacological intervention (e.g., SPT inhibition using Myriocin [12, 29–31] or L-cycloserine [32], DES inhibition using Fenretinide [33], CerS1 inhibition using P053 [34], CerS6 depletion using antisense oligonucleotides (ASO) [35]) can ameliorate high-fat diet-induced metabolic dysfunction. These studies have identified the critical contribution of de novo ceramide formation to the development and progression of obesity-associated metabolic diseases.

Once ceramides are produced, they can be transported within the cell and used for the generation of more complex sphingolipid species. On the ER-lipid droplet interface, long-chain-fatty-acid-CoA ligase (ACSL5) forms a multiprotein complex with the CerS enzymes and diacylglycerol acyltransferase (DGAT2) to catalyze ceramide acylation at the 1-hydroxy position [36]. This process appears to be relevant in the lipid-laden liver, possibly to divert ceramides from a bioactive- to a storage pool sequestered in lipid droplets in the form of less-toxic acylceramide species [36]. Ceramides in the ER are transported to the trans-Golgi apparatus at membrane contact sites through vesicular or non-vesicular pathways. Active transport involves the ceramide transport protein (CERT) that shuttles various types of species (C_14:0_–C_20:0_) to the Golgi apparatus for incorporation into sphingomyelin with lower efficacy for longer acyl chain ceramides [37–39]. Transport of ceramides to the cis-Golgi cisternae is required for glucosylceramide production, which are converted to more complex glycosphingolipids in trans-Golgi regions [40]. Complex sphingolipids can be enzymatically degraded to regenerate ceramides either via (a) *sphingomyelin hydrolysis* or (b) *sphingolipid salvage* [19]. These pathways involve (a) sphingomyelinases (SMase) to produce ceramides from sphingomyelin, and (b) ceramidases (CDase) that degrade ceramides obtained from sphingolipid catabolic breakdown to produce sphingosine that can be re-acylated to ceramides [41, 42]. Alternatively, sphingosine can be modified to sphingosine-1-phosphate (S1P), a potent sphingolipid signaling metabolite that is either dephosphorylated to regenerate sphingosine or irreversibly cleaved at the unique exit point of the sphingolipid-metabolic pathway [43]. Notably, targeted induction of ceramide degradation as achieved by tissue-specific overexpression of acid CDase or stimulation of ceramidase activity leads to beneficial metabolic outcomes in obese mice, similar to the inhibition of ceramide synthesis [44, 45]. Together, these studies have demonstrated the therapeutic potential of ceramide-lowering compounds in treating obesity-related metabolic diseases.

The enzymes involved in ceramide/sphingolipid turnover are active at distinct subcellular locations, with corresponding local differences in sphingolipid concentrations [19]. For example, members of the CerS family were detected in the ER, Golgi complex, mitochondria, and mitochondria-associated membranes (MAMs), while members of the SMase family show additional activity in the plasma membrane, nuclear envelope, and lysosome [16]. Critically, the subcellular localization of ceramides dictates their specific functions, and ceramide accumulation at spatially distinct sites in the cell produces specific metabolic outcomes (the reader is directed to a recent review on the organelle-specific regulation and function of ceramides [16]). Noteworthy is the complex regulation of endogenous ceramide turnover that depends on the availability of precursor substrates (amino acids and fatty acids) and is modulated by a number of intra- and extracellular stimuli (reviewed in [46]). In addition, three orosomucoid-like (ORMDL) proteins can sense ceramide levels in the ER membrane to cooperatively mediate feedback inhibition of de novo ceramide synthesis through interaction and modulation of SPT [47–51]. The complex regulatory network of ceramide turnover indicates that cellular ceramide levels need to be kept in a narrow range to maintain predetermined amounts for cellular integrity while ensuring rapid adaptation in ceramide concentrations in response to environmental changes and metabolic cues and preventing them from reaching cytotoxic levels.

## Metabolic roles, modes of action, and toxic features of ceramides

**Fig.2:**
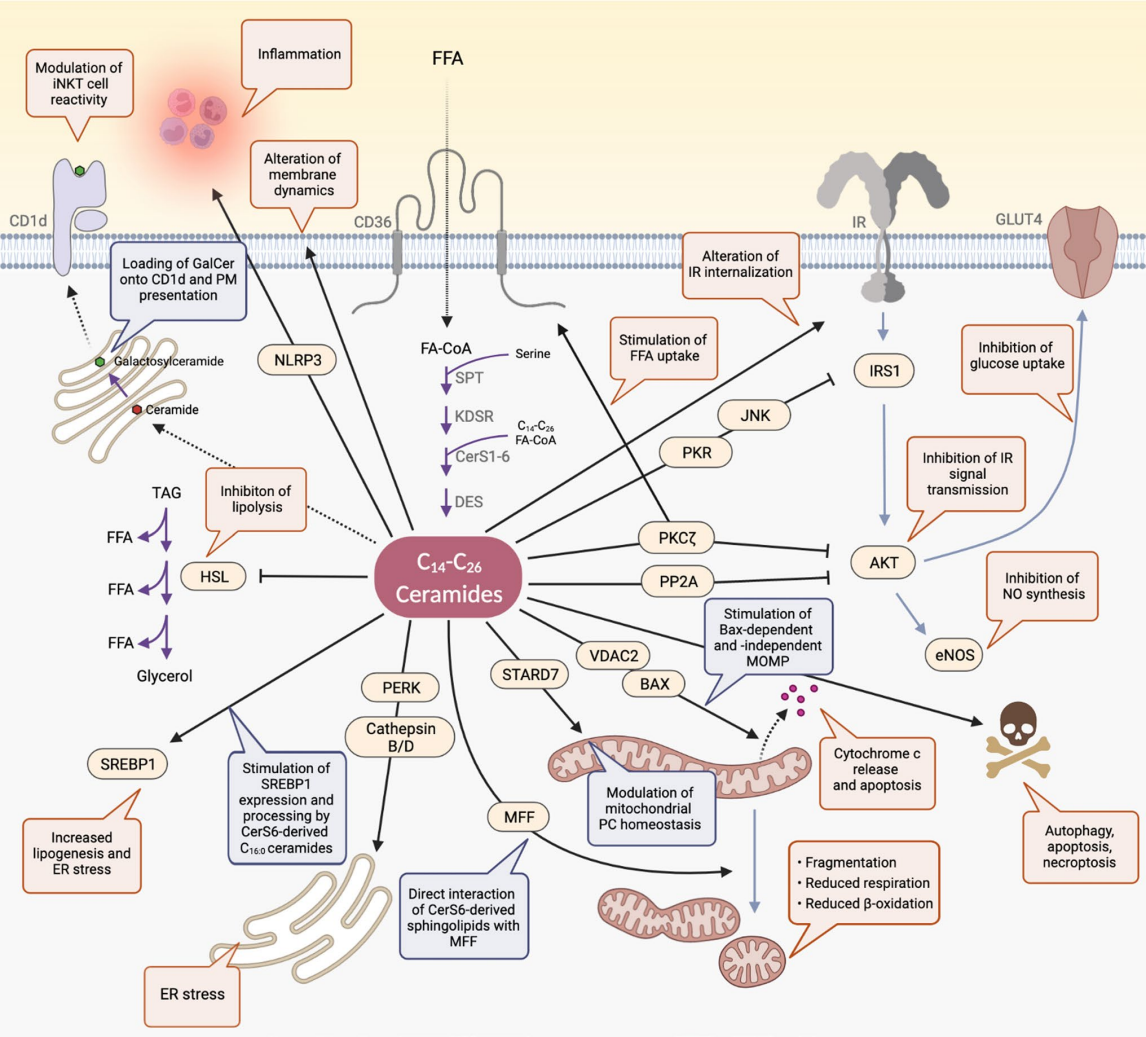
Cellular and molecular mechanisms by which ceramides affect metabolic regulation. Excessive influx of free fatty acid (FFA) mediated by the fatty acid transporter CD36 drives the production of ceramides, which exert multifaceted effects to modulate cellular metabolic homeostasis. Ceramide-dependent effects are shown by black arrows, including regulatory proteins through which they act. The consequences of ceramide accumulation are highlighted in red, and the underlying mechanisms are highlighted in blue. Purple arrows depict conversion of lipids, and dashed lines indicate transport. *AKT* protein kinase B, *BAX* Bcl-2-associated X protein, *CD1d* cluster of differentiation 1d, *CD36* cluster of differentiation 36 (fatty acid transporter), *CerS* ceramide synthase, *DES* dihydroceramide desaturase, *eNOS* endothelial NO synthase, *ER* endoplasmic reticulum, *FA-CoA* fatty acyl-coenzyme A, *FFA* free fatty acid, GalCer galactosylceramide, *GLUT4* glucose transporter 4, *HSL* hormone-sensitive lipase, *iNKT* invariant natural killer T cell, *IR* insulin receptor, *JNK* c-Jun-N-terminal kinase, *KDSR* 3-ketodihydrosphingosine reductase, *MFF* mitochondrial fission factor, *MOMP* mitochondrial outer membrane permeabilization, *NLRP3* NLR family pyrin domain-containing 3, *NO* nitric oxide, *PC* phosphatidylcholine, *PERK* protein kinase RNA-like ER kinase, *PKCζ* protein kinase C zeta, *PKR* protein kinase R, *PM* plasma membrane, *PP2A* protein phosphatase 2A, *SPT* serine palmitoyltransferase, *SREBP1* sterol regulatory element-binding protein 1, *STARD7* StAR-related lipid transfer protein 7, *TAG* triacylglycerol, *VDAC2* voltage-dependent anion channel 2

Different biological functions have been attributed to ceramides, and current research aims at assigning them to individual ceramide molecular species. In this context, it has already been indicated that ceramides with specific acyl chain lengths (C_16:0_ and C_18:0_) have a metabolic impact [52]. In contrast, other ceramide molecular species (C_24:0_) do not, but what accounts for this specificity remains ill-defined [52]. Most conclusions about the physiological roles of ceramides have been drawn from studies aimed at inhibiting ceramide build-up or increasing ceramide degradation under conditions of fatty acid oversupply (e.g., fatty acid treatment in cells or high-fat diet feeding in mice). Few studies targeted the overexpression of ceramide biosynthetic genes associated with increased ceramide formation. Studies in which cells or animal models were treated with artificial short-chain ceramide analogs (C_2_ and C_6_ ceramide) provided additional evidence in this context; and although such analogs do not match the physicochemical properties of naturally occurring ceramides, their sphingoid backbone is rapidly recycled and re-acylated to long-chain species of functional relevance [53]. Together, these studies have provided compelling evidence that ceramides act as cell-autonomous nutrient sensors that accumulate with increasing fatty acid concentrations to adjust lipid and glucose homeostasis (this theory is discussed in detail elsewhere [54]). The hypothesis of ceramides acting as metabolic messengers upon fatty acid excess through cell-type-specific responses is based primarily on the following observations: when plasma FFA levels rise, acyl-CoA concentrations increase in oxidative tissues, which can be readily re-esterified to ceramides. Here, ceramides affect membrane dynamics, and they modulate transmembrane signaling at specific intracellular sites in part through direct interaction with regulatory proteins [55, 56]. Thereby, ceramides diminish insulin signaling, presumably to adjust metabolic substrate storage and utilization according to the degree of fatty acid flux [57]. Ceramides regulate mitochondrial plasticity, respiration, and the capacity for β-oxidation in adipocytes, hepatocytes, and myocytes [58]. Additionally, ceramides can stimulate the cellular uptake of fatty acids [44]. At the same time, a ceramide-induced increase in hepatic de novo lipogenesis may support the incorporation of incoming fatty acids into glycerolipid pools, e.g., for their intermediate storage esterified in TAGs [25]. Ceramides also block lipolysis in adipocytes, restricting the further supply of fatty acids from endogenous stores [25]. Thus, under physiological conditions, ceramides may promote the utilization of fatty acids or their storage in non-toxic molecules to limit their lipotoxic effects, characterized by detergent-like activities [54]. Lastly, when the amount of integrated fatty acids reaches a predetermined physiological maximum, excessive ceramide accrual also in mitochondrial membranes initiates programmed cell death [59], which may reflect an inherent ability of organisms to limit the toxic effects of compromised cells. In this regard, it is tempting to speculate that the adverse metabolic effects of ceramide accumulation result from an adaptive response to increased FFA flux that fails under the chronic metabolic burden in obesity through dysregulation of ceramide metabolism, rather than from ceramide function per se. The existence of regulated feedback loops and a wide array of metabolic pathways that cooperatively maintain tight control of ceramide levels under normal conditions support this theory. In practice, however, prolonged intake of foods high in fat leading to obesity can result in abnormal accumulation of ceramides in both plasma and tissues, and chronic ceramide actions may become deleterious, causing degenerative conditions. A selection of the key findings on the physiological relevance and lipotoxic properties of ceramides in obesity are summarized in (Fig. 2) and discussed in more detail in the following section.

### Ceramides, biological membranes, and protein binding

Naturally occurring ceramides are of exceptional hydrophobicity and thus committed to cell membranes at low relative concentrations under basal conditions (< 1 mol%), but their levels significantly increase in response to fatty acid excess and other cellular stressors [55]. Hence, ceramide accumulation in the cell corresponds to alterations in membrane ceramide composition, and it is postulated that sustained ceramide excess in obesity impairs membrane dynamics [43]. Membrane ceramide enrichment may reduce membrane fluidity; this process is related to diabetes etiology [60, 61]. As mainly ascertained from monolayer- and bilayer-based model systems, ceramides can increase the molecular order of phospholipids, modulate the permeability of membranes to specific solutes, and promote lateral phase separation, transient nanodomain formation, and transmembrane (flip-flop) lipid motion (for more details, we refer the reader to [55]). Some of these properties stem from the ability of ceramides for intramolecular H-bonding in the polar region, which permits close ceramide packing in membranes [62]. Thereby, ceramides can assemble into raft-like ceramide-enriched membrane platforms (CEPs) to direct the recruitment, clustering, and activity of adaptors, receptors, and other signaling molecules [63]. Ceramides can also undergo polar headgroup interactions with other sphingolipid species [64]. A delicate balance of phase formation and transformation with mutual displacement of cholesterol by ceramides through interaction with sphingomyelin may exist, illustrating the interrelation of membrane sphingolipids and cholesterol and the multifaceted consequences resulting from imbalanced membrane ceramide plasticity [65, 66].

Several in vitro studies indicate that the length and saturation of the acyl chain define the biophysical characteristics of ceramide species, which may account for their differential effects on metabolic control [55]. For example, the membrane rigidifying and phase separation properties of ceramides are less pronounced for unsaturated compared to saturated molecular species, and the latter in particular have been linked to metabolic deregulation [67]. Interestingly, membranes isolated from tissues of CerS2-null mice show a variety of organ-specific changes in membrane fluidity, morphology, and trafficking, indicating that alterations at the level of selective ceramide synthesis in vivo can potently affect membrane dynamics [68]. These effects may underlie the detrimental phenotypes observed in CerS2-deficient mice, i.e., disturbed liver homeostasis, hepatopathy, hepatocarcinogenesis, and neurological abnormalities [69–71]. A follow-up study suggested that CerS2- versus CerS5-derived ceramides exert distinct effects on membranes [72]. Here, significant differences in the global order of the plasma membrane and CEP formation were observed in CerS2- versus CerS5-overexpressing HEK cells treated with bacterial SMase [72]. Thus, it has been concluded that ceramides with specific acyl chain lengths change the membrane properties to different extents. Still, the precise roles of the acyl chain ceramide distribution in membranes of eukaryotic cells and its consequences on metabolic regulation in obesity require further investigation. Another consideration is that cells exhibit a variety of different membranes, characterized by unique features through specific lipid and protein composition and specific interactions of the two [73, 74]. Furthermore, ceramide sub-compartmentalization and local changes in membrane ceramide concentrations are expected to be crucial factors in executing both their biological functions and pathological effects. This is also exemplified by a recent study on C_16:0_ ceramides, which impair mitochondrial dynamics in the mouse liver and systemic glucose metabolism when they accumulate in mitochondrial membranes and MAM in obesity [26].

In addition, ceramides act through direct interaction with and modulation of membrane proteins. Initial studies have identified proteins that target a mixture of ceramides, e.g., in protein-lipid overlay assays in vitro. Nevertheless, it must be considered that the physiological environment, the subcellular localization, and the acyl chain length confer specificity for certain sphingolipid–protein interactions in the cell [75]. We have recently demonstrated this using a sphingolipid precursor probe, i.e., a photoactivatable and clickable sphingosine molecule (Ref. [76]), which allowed co-precipitating sphingolipids with their protein-binding partners in cultured HeLa cells and those deficient in CerS5- or CerS6-dependent C_16:0_ ceramide synthesis [26]. These experiments revealed previously unknown protein targets of sphingolipids depending on C_16:0_ ceramide formation and yielded distinct protein targets of CerS5- versus CerS6-derived ceramides, a specificity likely due to differences in their spatial distributions [26]. In the future, it will be important to uncover the acyl chain length- and cell compartment-specific protein interactome of ceramides to provide a more detailed picture of the ceramide-dependent regulatory networks of cellular and systemic metabolism. Understanding the physicochemical properties of ceramides in relation to their biological functions is crucial for better understanding their pathological implications, which are likely to occur as a result of ceramide-dependent changes in membrane homeostasis and protein-ceramide interactions.

### Ceramides, insulin signaling and glucose homeostasis

Obesity results in the deregulation of several cell-intrinsic pathways partly due to increased fatty acid influx and ceramide build-up, thus impairing insulin signal transmission [57]. Evidence indicates that ceramides directly interfere with the insulin receptor (IR) signaling cascade [57]. In insulin-responsive cells, insulin-binding triggers IR autophosphorylation that recruits IR substrates (IRS) to induce a downstream response leading to phosphorylation and activation of the protein kinase B/AKT [77]. AKT modulates various downstream regulatory proteins to confer a pro-survival signal while promoting nutrient uptake and anabolic metabolism [77]. The insulin-desensitizing properties of ceramides were first noted in cultured adipocytes, (cardio)myocytes, and hepatocytes, wherein the treatment with short-chain ceramide analogs led to inhibition of the insulin-stimulated phosphorylation of AKT, similar to the effect observed upon treatment with the saturated fatty acid palmitate that primarily fuels C_16:0_ ceramide de novo production [33, 78–83]. Indeed, ceramides induce a wide range of their metabolic effects at the level of AKT through distinct mechanisms involving the serine/threonine protein phosphatase 2A (PP2A) and the atypical protein kinase C zeta (PKCζ). It has been postulated that in adipocytes, myotubes, and vascular smooth muscle cells, ceramides direct PKCζ to caveolin-enriched microdomains (CEMs) to sequester AKT in a repressed state [84, 85]. In cells with a lower abundance of CEMs, ceramides within the plasma membrane promote dephosphorylation of AKT by activating PP2A [86, 87], but both pathways may co-exist within the same cell type [88]. By modulating AKT activity, ceramides also interfere with plasma membrane translocation and fusion of the GLUT4 glucose transporter in adipocytes and myocytes, suggesting that ceramides take critical roles in deregulating glycemic control by influencing insulin-dependent glucose uptake into adipose tissue and muscle [78, 89]. Furthermore, sustained ceramide action in cultured myocytes triggers JNK-dependent inhibitory phosphorylation of IRS1 via RNA-activated protein kinase (PKR) and may modify IR translocation into membrane lipid microdomains in control of insulin sensitivity [90, 91].

The transient influx of fatty acids into insulin-target tissues can diminish insulin signaling presumably as an adaptive response to adjust metabolic substrate handling, e.g., for the mobilization of energy stores in times of nutrient deficiency. Ceramides, formed from the incoming fatty acid sources, likely mediate these effects. Accordingly, it is interesting to postulate that the inhibitory effects of ceramides on insulin signal transmission are a relevant process in the insulin-dependent regulation of glucose and lipid metabolism through adaptive insulin resistance. However, such an adaptive response to transient fatty acid supply quickly becomes maladaptive upon prolonged fatty acid excess during obesity development, contributing to sustained reductions in IR signaling [92]. A critical role of ceramide accumulation in insulin-target tissues during obesity development and its link to the manifestation of insulin resistance in vivo has been mainly determined by the rate of insulin-stimulated AKT phosphorylation in key metabolic tissues of rodent models after ceramide-lowering interventions. For example, inhibition of SPT using Myriocin treatment to reduce global ceramide synthesis improved the ability of insulin to stimulate AKT in the liver and skeletal muscle of genetically obese (ob/ob) or diet-induced obese mice [29] and in the gastrocnemius muscle of obese and diabetic db/db mice [30]. Although this does not provide direct proof that ceramide accumulation alone is sufficient to attenuate insulin signal transmission, it indicates the insulin-sensitizing effects of limiting ceramide build-up in obesity.

More recently, a particular role of C_16:0_ ceramides in the regulation of insulin sensitivity and glucose homeostasis has been identified [26, 27, 35, 93]. As discussed in more detail below, CerS6-dependent C_16:0_ ceramide production is increased in specific tissues during obesity development, and transgenic expression of CerS6 in primary hepatocytes is sufficient to inhibit insulin-stimulated phosphorylation of AKT [27, 93]. Conversely, body-wide ablation of CerS6-derived C_16:0_ ceramide synthesis in mice fed a high-fat diet improved insulin-evoked AKT phosphorylation in the liver and in palmitate-treated primary hepatocytes isolated from mice with liver-specific CerS6 deficiency [27]. However, whether the effects on insulin sensitivity in these murine models are attributable to direct modulation of the IR signaling cascade, whether they occur secondary to changes in other cellular processes, or the combination of both, remains to be carefully differentiated. This distinction must also be considered in light of current discussions that simple, unitary defects in proximal insulin signaling may not be the primary cause of systemic insulin resistance in type 2 diabetes [94]. Undoubtedly, additional challenges that cooperatively compromise cellular homeostasis and trigger cellular stress with multifaceted effects on insulin sensing and signal transmission must be taken into account.

### Ceramides and lipid homeostasis

Ceramides contribute to the homeostatic control of lipid metabolism by modulating the uptake, storage, and oxidation of fatty acids in adipocytes, hepatocytes, and myocytes. This appears to be attributable also to insulin-independent processes. Thus, blocking general ceramide synthesis in mouse liver through deletion of *Degs1* improved hepatic insulin sensitivity but markedly reduced the expression of the sterol regulatory element-binding protein (*Srebf1*) mRNA and a variety of its downstream targets that control de novo lipogenesis [25]. It has recently been proposed that ceramides activate lipogenesis in the liver by modulating the activity of the SREBP1 protein and that CerS6-derived C_16:0_ ceramides are particularly relevant to this process [95]. In this way, (C_16:0_) ceramides may contribute to the selective insulin resistance paradox, wherein the insulin-resistant liver fails to suppress glucose production but continues to stimulate lipogenesis, which is a central mechanism in the pathophysiology of hepatic steatosis and type 2 diabetes [96, 97]. Similarly, ceramides stimulate the cellular uptake of fatty acids via PKCζ-mediated CD36 plasma membrane translocation in hepatocytes and adipocytes, where incoming fatty acids may be a direct source of both de novo lipogenesis of TAGs and ceramide synthesis once the former process is saturated [24, 25, 44]. In primary adipocytes, C_2_ ceramide treatment attenuated the stimulatory effects of β-adrenergic agonism on hormone-sensitive lipase (HSL) phosphorylation and activation, indicating that ceramide action can also inhibit lipolysis [25]. Furthermore, ceramides regulate the cellular capacity to oxidize incoming fatty acids in obesity [27, 34, 93]. Specifically, a reduction in C_16:0_ ceramide levels due to CerS6 deficiency in liver or BAT of mice increases mitochondrial β-oxidation capacity during high-fat diet feeding [27], whereas an increase in C_16:0_ ceramide levels in CerS2-haploinsufficient mice impairs hepatic β-oxidation [93]. Together, these studies revealed a critical inhibitory role of CerS6-derived C_16:0_ ceramides in the liver and BAT for β-oxidation. In contrast, in the skeletal muscle of obese mice, partial inhibition of CerS1-dependent C_18:0_ ceramide synthesis was sufficient to increase mitochondrial β-oxidation in myocytes, indicating the tissue-specific effects of ceramide species to control fatty acid turnover [34].

### Ceramides and mitochondrial efficacy

Mitochondria play a central role in energy homeostasis, and defects in mitochondrial integrity are associated with obesity-related diseases such as heart failure, fatty liver disease, and diabetes [98, 99]. Obese individuals often exhibit altered mitochondrial morphology and diminished mitochondrial function in oxidative tissues, in part due to mitochondrial lipid accumulation [100, 101]. Ceramides can be detected in mitochondrial membranes, and it has become evident that certain ceramide species interfere with mitochondrial integrity [58].

The origin of mitochondrial ceramides is not entirely clear, but there is emerging evidence for mitochondria-autonomous ceramide production pathways [102]. Enzymes involved in ceramide turnover, including members of the CerS, SMase, and CDase families, co-localize with common mitochondrial markers or have been co-purified with mitochondria from cell or tissue extracts, suggesting that mitochondrial ceramides originate from different intraorganellar processes [103–105]. Furthermore, CerS activity has been detected in both inner and outer mitochondrial membranes, and CerS isoforms interact differentially with inner and outer membrane proteins, suggesting sub-organellar differences in ceramide synthesis [106, 107]. Efficient intramitochondrial de novo ceramide production has recently been corroborated by the observation that a subfraction of SPT localizes to the ER-mitochondrial interface to modulate mitochondrial ceramide content [108]. While a significant fraction of SPT is formed by SPTLC1 and SPTLC2 cis-assembly in the ER membrane, a portion of SPTLC2 is detectable in the mitochondrial outer membrane where it interacts in *trans* with ER-localized SPTLC1 at mitochondria-ER contact sites, possibly to provide 3-ketosphinganine for subsequent mitochondrial ceramide formation [108]. DES1 and KDSR also exhibited dual localization to the ER and mitochondria, arguing for a mitochondria-autonomous ceramide de novo synthesis machinery [108]. However, it cannot be excluded that the MAM is a major site for ceramide production to ensure mitochondrial ceramide supply. Enzymes required for ceramide biosynthesis can also be detected in MAM, and additional steps of lipid synthesis in MAM and MAM-to-mitochondria ceramide transport may co-exist [106].

Mitochondrial ceramides can modulate respiratory capacity in different oxidative tissues. As such, treating rat skeletal muscle mitochondria with different ceramide species impairs the ability for oxidative phosphorylation of ADP [109]. Conversely, reducing CerS6-derived mitochondrial C_16:0_ ceramides in the liver of obese mice increased ADP-stimulated mitochondrial respiration [26]. In some early seminal studies, ceramides were thought to directly interfere with components of the mitochondrial electron transport chain, modulating respiration and elevating the production of reactive oxygen species (ROS), with deleterious metabolic consequences [110–112]. These assumptions were based primarily on findings in cultured cells in which short-chain ceramide analog treatment inhibited mitochondrial respiratory chain complexes I and III [112, 113]. In addition, complex IV activity was inhibited upon incubation of mitochondria isolated from mouse liver with C_16:0_ ceramides but not upon incubation with C_24:0_ or C_24:1_ ceramides, arguing for ceramide species-specific effects on mitochondrial respiratory function [114]. A reduction in complex IV and II activity was also observed in the liver of mice with CerS2-haploinsuffiency, wherein C_16:0_ ceramides accumulate [93]. However, despite the evidence implicating C_16:0_ ceramides in the direct impairment of mitochondrial respiratory complex function, mice protected from the obesity-associated increase in C_16:0_ ceramides exhibited elevated mitochondrial respiration in the liver and BAT, albeit with a slight reduction in complex IV activity and without changes in other components of the respiratory machinery [27]. The above suggests that direct effects on electron transport chain components may not be the primary mechanism at work for (C_16:0_) ceramides to impair mitochondrial function in obesity, but alternative mechanisms may be at play through which ceramides secondarily alter mitochondrial respiration.

It has been found that ceramides can modulate mitochondrial morphology through direct interaction with the fusion/fission machinery of mitochondrial membranes [26]. Transient morphological changes are necessary for a dynamic adaptation of mitochondrial (respiratory) function to a variety of metabolic cues, also to balance intracellular fuel utilization and partitioning [115–117]. It was initially observed that fatty acid turnover in cultured cells triggers mitochondrial fragmentation through increased de novo ceramide synthesis [118]. Similarly, treatment with ceramide analogs promoted mitochondrial fragmentation in (cardio)myocytes [118, 119] and disrupted mitochondrial function in INS-1 β-cells [120]. Intriguingly, CerS6 and its derived C_16:0_ ceramides localize to and accumulate in hepatic mitochondria and MAM in obesity, promoting mitochondrial fragmentation in the liver of mice, diminished mitochondrial respiratory capacity, and defective glucose handling [26]. This occurs through a direct interaction of sphingolipids derived from CerS6-dependent ceramide formation with mitochondrial fission factor (MFF), an adaptor protein critical for mitochondrial membrane translocation of dynamin-related protein (DRP1) and the initiation of membrane fission [26]. Interestingly, MFF exhibits binding specificity towards CerS6-derived sphingolipids versus diacylglycerols (DAGs), which are also associated with mitochondrial fission events and are known to promote insulin resistance, but likely via a different mechanism [121–123]. Similar to CerS6, *Sptlc2*-deficient cells are protected from palmitate-induced mitochondrial fragmentation, which is partially restored upon re-expression of a mitochondria-directed but not an ER-directed SPTLC2 variant, suggesting a role of palmitate-driven de novo ceramide production at the ER-mitochondria junction in this process [108].

Moreover, it has been found that ceramides can bind STARD7, which acts as an intramitochondrial lipid transfer protein for phosphatidylcholine (PC) to shuttle PC between outer and inner membranes, and thus is involved in the dynamic regulation of mitochondrial lipid composition [56, 124]. PC concentrations in the inner membrane are important for the maintenance of respiration and cristae morphogenesis, and deficiencies in intramitochondrial PC transport can have profound effects on mitochondrial membrane homeostasis [124]. Changes in mitochondrial ceramide content could therefore lead to broader alterations in mitochondrial lipid plasticity to regulate mitochondrial respiration and alternative functions, a hypothesis that warrants further investigation.

The mitochondria-related effects of ceramides are likely driven not only by actions in mitochondrial membranes but also by impact within other cellular compartments such as MAMs, which are closely linked to the control of mitochondrial function. In the MAM, ceramides affect protein incorporation and MAM functionality [125, 126]. Disruption of MAM integrity, in turn, triggers metabolic inflexibility, insulin resistance, and cellular dysfunction in tissues [127], which may in part result from the obesity-related accumulation of ceramides at this particular subcellular site.

### Ceramides and ER stress

The ER is the primary site for ceramide biogenesis involved in numerous metabolic processes, including calcium storage, lipid biosynthesis, and protein folding, while being vulnerable to lipotoxicity. Given the physicochemical properties of ceramides, it is likely that alterations in ceramide turnover affect ER ceramide content and subsequently ER membrane homeostasis, but not much is known about this relationship [128]. Ceramide-dependent control of ER proteostasis has been demonstrated in yeast, with specific acyl chain length ceramides regulating the sorting of GPI-anchored proteins into selective export sites of the secretory pathway [129]. Notably, any disturbance in ER proteostasis can induce the unfolded protein response (UPR) as a protective mechanism to restore internal homeostasis, but this response is insufficient to recover ER functionality in peripheral tissues and the brain in obesity leading to sustained ER stress and metabolic deterioration [130–132]. It is postulated that ceramides play a critical role in this process [133]. As such, ceramides promote ER stress and insulin resistance in the liver of mice with alcoholic [134] and non-alcoholic fatty liver disease [95], as well as in the hypothalamus during obesity development [135], associated with adverse metabolic consequences. In yeast, increased ceramide production through inhibition of the negative feedback regulation of SPT triggers chronic UPR activation and impairs ER-to-Golgi transport [136]. Moreover, in mammalian cultures, the UPR transducer ATF6 can be activated through direct interaction with two intermediates of ceramide synthesis, namely sphinganine and dihydroceramide, involved in physiological settings that show ER membrane expansion [137]. However, only few mechanisms have been proposed in vitro for ceramides to directly interfere with ER stress modulators, including C_16:0_ ceramide-dependent binding and activation of cathepsin B/D and activation of CD95-PERK signaling [133, 138, 139]. Still, the relevance of these pathways to ceramide-depended ER stress in the context of obesity remains elusive.

The availability of fatty acids for the synthesis of ceramides with specific acyl chain lengths determines the effects of ceramides on ER homeostasis. In mouse hepatocytes, palmitate (C_16_)-dependent increases in ceramide content were associated with increased expression of UPR marker genes, which was potentiated by the addition of myristate (C_14_), and reversed by inhibition of de novo ceramide synthesis [140]. The observation that myristate but not palmitate stimulated ER stress in intestinal epithelial cells through increased expression of CerS5 and CerS6 and increased C_14:0_ ceramide synthesis, supported the notion of cell-type-specific regulation and function for ceramides in this process [141]. Along these lines, in Hep3B cells, CerS6- but not CerS5-dependently formed C_16:0_ ceramides promoted ER stress, while CerS2-dependently formed longer-chain ceramides (C_22:0_–C_24:0_) elicited a protective effect [95].

The consequences of ceramide accumulation within the ER membrane are poorly understood. In yeast, it has been demonstrated that ceramide transfer out of the ER through increased ER-Golgi tethering during ER stress prevents the lipotoxic effects of ceramides on ER integrity [142]. From a different perspective, palmitate treatment impaired ceramide flow from the ER to the Golgi apparatus in insulinoma cells, promoting ER stress [143]. Similarly, blocking ER-to-Golgi ceramide traffic by inhibiting CERT in cultured myocytes potentiated the deleterious actions of lipotoxicity on insulin signaling [144]. However, it is unclear whether these effects result from ceramide accumulation in the ER membrane or insufficient availability of ceramides for the synthesis of complex sphingolipids in the Golgi apparatus, a matter that requires further investigation. Moreover, while most studies have linked ceramides to the induction of ER stress, evidence from in vitro experiments suggests that C_16:0_ ceramides may play a protective role in certain cell types. For example, it was reported that the generation of C_16:0_ ceramides by CerS6 protected human head and neck squamous cells from ER stress, whereas knockdown of CerS6 and a subsequent decrease of C_16:0_ ceramide content induced ATF6 expression via perturbation of ER Ca^2+^ homeostasis, which disrupted ER-Golgi networks leading to ER stress [145, 146].

### Ceramides and inflammation

Obesity is accompanied by chronic inflammation in several tissues, which triggers adverse effects on insulin sensitivity. A mechanism of ceramide-induced lipotoxicity involves the NLRP3 inflammasome that can sense intracellular ceramides in adipose tissue and macrophages to induce inflammatory signaling and insulin resistance [147]. *Nlrp3*-deficient animals are protected from obesity-associated hepatic steatosis, adipose tissue inflammation, and glucose intolerance, supporting the notion that the effects of ceramides on NLRP3-dependent pathways may be relevant in the etiology of these metabolic disorders [147]. Further evidence indicates that ceramides affect signaling of tumor necrosis factor (TNFα) in control of inflammation and apoptosis, as disruption of membrane lipid microdomains in CerS2-null mice prevented the internalization and downstream signaling of the TNFα receptor (TNFR) [148].

Ceramides in antigen-presenting cells may further regulate the activity of particular immune cells. Invariant natural killer T (iNKT) cells, which are highly enriched in white adipose tissue, can react to lipid antigens presented in CD1d molecules with profound immunomodulatory potential [149]. In particular, glycosylated sphingolipids can be loaded onto CD1d and presented at the plasma membrane providing a potent ligand for iNKT cell activation [150]. α-galactosylceramide, a synthetic prototype iNKT cell lipid antigen derived from structure–activity relationship studies of its natural analog, was found to ameliorate the metabolic defects associated with a high-fat diet in mice [151]. Rates of endogenous ceramide turnover may thus result in alterations of endogenous glycosphingolipid pools, interfering with iNKT cell modulation and metabolic control. It is proposed that adipose tissue-resident iNKT cells exert protective roles in the development of obesity-associated diseases through regulatory cytokine production and stimulation of macrophage polarization [151, 152]. Yet, another study found that iNKT cells contributed to tissue inflammation, insulin resistance, and hepatic steatosis [153]. Notwithstanding these conflicting data, it is tempting to speculate that the pathophysiological properties of ceramides in immunometabolic diseases involve a role in providing iNKT cell lipid antigens.

Although several studies have uncovered cell-autonomous effects of ceramides on immune cell homeostasis, such a role in obesity-related diseases remains unclear [154]. De novo ceramide synthesis in cultured macrophages interferes with autophagosome formation, a process thought to play a critical role in regulating innate immunity [155, 156]. However, deleting either *Sptlc2*, *Degs1,* or *CerS6* in the myeloid lineage in vivo did not result in metabolic alterations in high-fat diet-fed mice [23, 25, 27, 157]. These findings suggest that ceramide accumulation in myeloid cells, at least owing to increased de novo ceramide synthesis, may not be the primary mechanism in the manifestation of obesity-related metabolic diseases.

### Ceramides and cell death

The role of ceramides in cell fate determination is probably the best-studied mechanism of ceramide action and has been extensively reviewed by experts in the field (e.g., [158–160]). Increased lipid-induced apoptosis (lipoapoptosis) often accompanies obesity and can be induced by ceramides in several cell types, leading to insulin resistance and metabolic dysfunction [161, 162]. Pro-apoptotic pathways that employ ceramides as second messengers appear to play an essential role in β-cell-, hepatocyte-, and cardiomyocyte death in the pathogenesis of type 2 diabetes mellitus, non-alcoholic fatty liver disease, and heart failure [163–165].

Ceramides have been linked to pro-apoptotic processes such as Fas-capping [166] and emerged as positive modulators of JNK- [167], kinase suppressor of Ras (KSR)- [168], and cathepsin D signaling [169] in stress-induced apoptosis. More recently, knockdown of selected CerSs revealed that a specific pool of C_16:0_ ceramides derived from CerS6 controls key events in the execution phase of apoptosis, such as the loss of Focal Adhesion Kinase (FAK) and permeabilization of the plasma membrane by regulating caspase-7 activity [170]. Ceramide-induced apoptosis has also been implicated in the activation of PKCδ, as the treatment with ceramide analogs induced PKCδ Golgi complex-translocation and apoptosis [171].

Moreover, ceramides evolved as important regulators of the mitochondria-intrinsic apoptotic pathway, with ceramide concentrations in mitochondria dictating apoptosis in cultured cells. Jain and colleagues have shown this in an elegant study where a mutated form of CERT (mitoCERT), which carries a mitochondrial anchor to facilitate ER-to-mitochondria ceramide transport, induced BAX-dependent mitochondrial outer membrane permeabilization (MOMP), cytochrome c release, and apoptosis [172]. This study confirmed earlier findings that BAX-dependent release of mitochondrial cytochrome c could be efficiently induced by ceramides, potentially in mitochondrial CEPs, and in particular by CerS6-derived C_16:0_ ceramides [173–176]. A proposed mechanism for ceramide-mediated apoptosis involves the interaction of ceramides with the voltage-dependent anion channel VDAC2 that provides a mitochondrial platform for BAX/BAK translocation [177]. Conversely, both disruption of ceramide synthesis and removal of ceramides from mitochondria via expression of a mitochondria-targeted CDase could prevent apoptotic processes [172]. Although still in debate, it has been suggested that MOMP also results from self-assembled ceramide pores (as shown for C_2_ and C_16:0_ ceramides) [178, 179]. The formation of such pores is inhibited by the incorporation of C_22:0_ ceramides that compete with C_16:0_ ceramides to form smaller channels to control the selective export of mitochondrial pro-apoptotic proteins and differential regulation of apoptosis [180].

In addition, CerS1-derived C_18:0_ ceramides bind LC3B-II at the outer mitochondrial membrane upon DRP1-mediated mitochondrial fission and direct autophagolysosomes to mitochondria to induce lethal mitophagy [181]. In the same study, exogenously applied C_16:0_ ceramides also localized to mitochondria, where they decreased mitochondrial oxygen-consumption rate and induced mitophagy [181]. However, it is unclear whether a specific threshold for ceramide concentration in mitochondrial membranes must be reached and what exactly determines the differential effects on mitochondrial respiratory function versus mitochondria-dependent death processes. Also, while most studies have shown that C_16:0_ ceramides act pro-apoptotic, it has been suggested that they trigger anti-apoptotic signals in certain other cell types [145]. More recently, it was found that C_16:0_ ceramides interact with RIP-kinase (RIPK1) in structures referred to as ceramidosomes, which assemble in the ER and translocate to the plasma membrane to trigger necroptotic signaling [182]. Together, these findings demonstrate the multifaceted effects of ceramides on different pathways leading to cell death.

## Obesity-induced alterations to ceramide metabolism

**Fig. 3:**
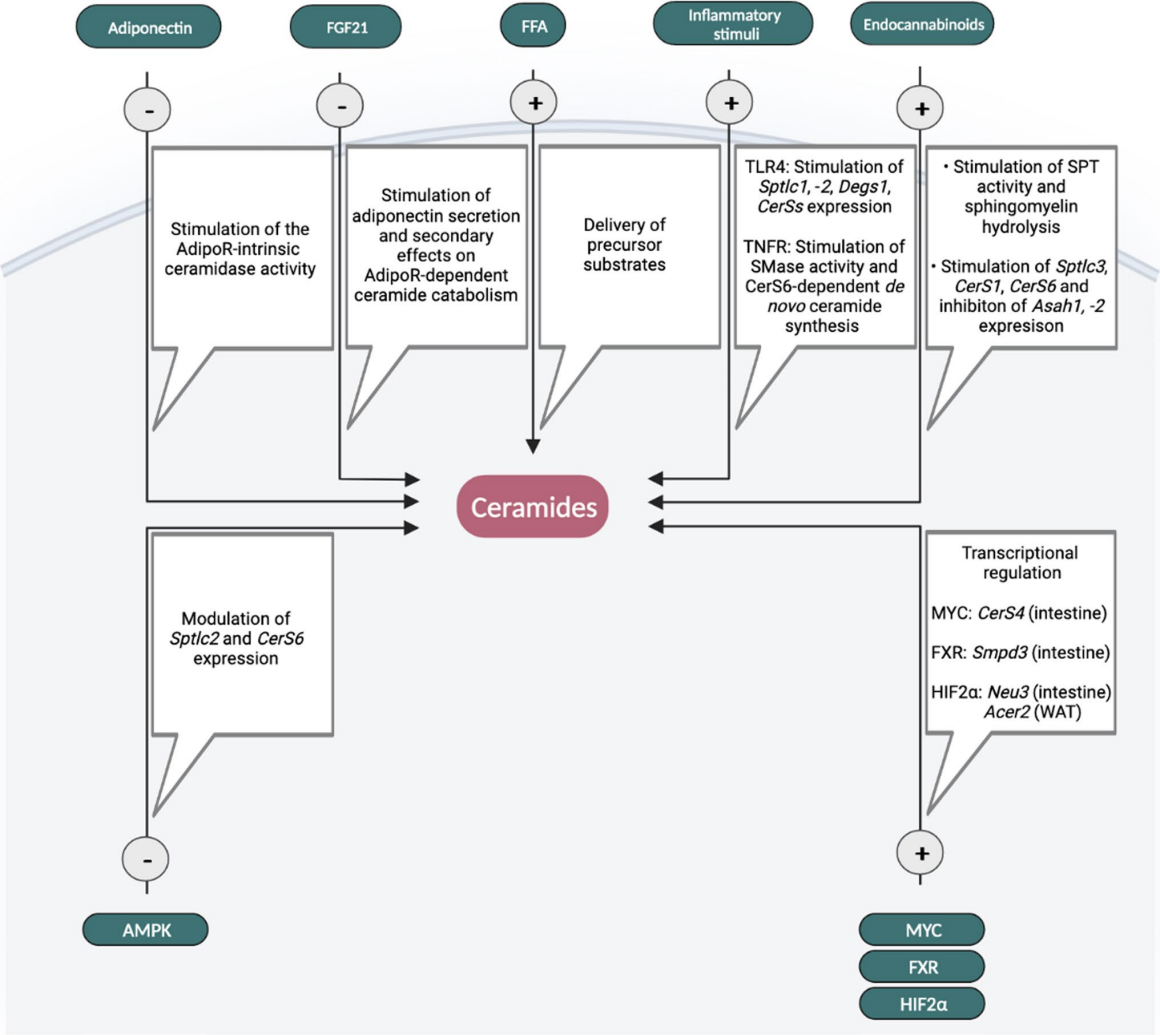
Factors potentially contributing to ceramide accumulation in obesity. In conjunction with the increased availability of precursor fatty acids for ceramide production, several cell-extrinsic and -intrinsic factors have been linked to the control of ceramide turnover rate and may contribute to ceramide accumulation when deregulated in obesity. *AMPK* AMP-activated protein kinase, *Asah* N-acylsphingosine aminohydrolase (acid CDase), *Acer2* alkaline ceramidase 2, *CerS* ceramide synthase, *FFA* free fatty acids, *FGF21* fibroblast growth factor 21, *FXR* farnesoid X receptor, *HIF2α* hypoxia-induced factor 2α, *MYC* transcription factor MYC, *Neu3* neuraminidase 3, *Smpd3* sphingomyelin phosphodiesterase 3 (neutral SMase2), *Sptlc* serine palmitoyltransferase long-chain base subunit

In obesity, alterations in endogenous ceramide turnover due to increased substrate availability and deregulations in the metabolic pathways that fine-tune ceramide synthesis under healthy conditions lead to the accumulation of ceramides in body tissues and circulation, thereby disrupting cellular function and metabolic integrity. Some of the pathways associated with modulation of ceramide turnover thought to promote ceramide accumulation in obesity are discussed in the following section (Fig. 3).

### Diet and substrate availability

A critical factor in tissue ceramide build-up is the diet providing precursor substrates, such as fatty acids, for endogenous ceramide formation. Dietary ceramides and complex sphingolipids are readily degraded in the intestinal tract, but their degradation into metabolites such as palmitate and serine can fuel tissue ceramide synthesis [183, 184]. Dietary sphingosine sources in turn can be directly used by the intestinal microbiota for the generation of sphingolipids, which can enter host circulation and routed to organs such as the liver for ceramide generation, thus impacting tissue ceramide content [183, 185]. The dietary fatty acid composition appears to determine the extent of ceramide formation and the related effects on body metabolism. In humans, it was found that diets rich in saturated fat increase plasma ceramide levels more than polyunsaturated fat, which has been associated with the development of liver steatosis and insulin resistance [186, 187]. Dietary effects on the gut microbiome also affect the levels of circulating ceramides linked to cardiovascular disease risk [188].

A large proportion of the fatty acids used for endogenous ceramide formation in obesity derives from adipose tissue lipid spillover. In obesity, increased adipocyte lipolysis as a result of inflammation and insulin resistance in adipose tissue promotes lipid mobilization from fat stores, increasing circulating FFAs and ectopic tissue influx that continuously supplies substrates for ceramide synthesis [189]. The ability of cells to import fatty acids and the availability of specific precursor fatty acids to promote the synthesis of ceramides with specific acyl chain lengths thus likely dictate the rates of endogenous ceramide production.

### Inflammatory signaling

Ceramides can induce an inflammatory response, promoting ceramide biogenesis in a vicious cycle of ceramide production and inflammatory signaling that causes systemic defects in glucose handling [190]. This is mediated in part via the toll-like receptor (TLR)4, a pattern recognition receptor that modulates innate immune responses and insulin sensitivity [190, 191]. Stimulation of TLR4 was found to trigger increased expression of *Sptlc1, -2*, *Degs1,* and specific *CerSs* in myocytes following stimulation with lipopolysaccharide or palmitate, indicating that TLR4 induces ceramide synthesis upon inflammatory input and fatty acid excess [192]. Accordingly, infusion of lard oil in mice increased ceramide levels in the liver, muscle, and hypothalamus, depending on the TLR4/IKK-β pathway [192]. Intriguingly, while the increase in ceramide formation was not required for TLR4-dependent induction of inflammatory cytokines, it was essential for TLR4-dependent insulin resistance, linking lipid signaling induced by inflammatory stimulation to decreased insulin action [192]. Conversely, mice deficient in TLR4 are protected from the lipotoxic effects of ceramides on insulin sensitivity [192]. Upon activation, TLR4 recruits the innate immune signal transduction adaptor MyD88, which is also involved in the signaling pathway of the inflammatory cytokine Interleukin-1beta (IL-1β). Accordingly, MyD88 has been associated with increased ceramide production following IL-1β stimulation in cultured hypothalamic neurons, depending on the activation of neutral SMase [193]. Similar observations were made for alternative pro-inflammatory-and-death signals (e.g., TNFα, Fas, and TRAIL), which mediate their cellular effects in part by stimulating ceramide formation [194–196]. Previous studies found that TNFα induces ceramide accumulation via coordinated changes in the ceramide de novo and sphingomyelin hydrolysis pathways [197]. It was suggested that TNFR stimulation independently activates acid- and neutral SMase by different cytoplasmic domains, specifically coupled to selected pathways of TNFR signaling [198]. More recently, a pronounced effect of TNFα treatment on C_16:0_ ceramide formation was identified in MCF-7 cells, which was inhibited by silencing CerS6 but not CerS5 [170]. This finding suggests a putative mechanism affecting specific ceramide pools upon increased inflammatory input in obesity.

### Adiponectin receptors

The adipokine adiponectin is predominantly secreted from mature white adipocytes and acts on several target tissues to exert anti-diabetic, anti-inflammatory, and cardioprotective actions [199]. Globular adiponectin expression in mouse models of obesity or atherosclerosis can ameliorate their detrimental cardiometabolic phenotypes by improving insulin sensitivity and inhibiting the progression of atherosclerotic lesions [200]. It has turned out that adiponectin exerts a large proportion of its beneficial properties via receptor-stimulated catabolism of tissue ceramides [45]. Adiponectin receptors (AdipoR1 and AdipoR2) possess intrinsic ceramidase activity, which is efficiently stimulated upon ligand-binding by 20-fold [45, 201]. AdipoR2 is capable of hydrolyzing shorter (C_6_) and longer (up to C_24_) ceramide substrates but appears to show a preference for C_18_ ceramide species [201]. In conjunction with these studies, inducible overexpression of AdipoR or oral administration of an AdipoR agonist (AdipoRon) can activate ceramidase activity to reduce tissue ceramide content and ameliorate diabetic phenotypes in mice, indicating its potential as a ceramide-lowering compound also in the treatment of obesity-associated metabolic diseases [202–204]. Similarly, the stress-inducible hormone fibroblast growth factor (FGF21) partly acts via a mechanism that involves adiponectin production and secretion, stimulating AdipoR-dependent ceramide degradation to enhance insulin sensitivity in multiple target tissues [205]. These findings further suggest that reduced circulating adiponectin levels in obesity may contribute to tissue ceramide accumulation by reduced stimulation of AdipoRs and insufficient ceramide degradation.

### AMPK

AMP-activated protein kinase (AMPK) is an energy-sensing enzyme that controls a variety of physiological events to maintain energy homeostasis, including glucose and lipid metabolism [206]. When cellular energy levels are low, AMPK activity increases to induce catabolic pathways while inhibiting anabolic routes to replenish cellular ATP. Stimulation of AMPK activity improves insulin sensitivity, and sustained decreases in AMPK activity in obesity are associated with insulin resistance [207]. It is predicted that AMPK inhibits ceramide synthesis to modulate insulin sensitivity and glucose homeostasis. Specifically, it has been shown that AMPK activation attenuates the palmitate-dependent increase in *Sptlc2* and *CerS6* expression and cellular ceramide content in cultured myotubes, but the mechanisms of how AMPK activity would affect the expression of these genes have not been addressed [208]. In turn, chronic activation of AMPK decreases de novo ceramide formation and reduces ceramide content in soleus muscle of high-fat diet-fed rats and palmitate-treated cultured astrocytes [208, 209].

More recently, it was found that hyperthyroid rats exhibit reduced ceramide content in the hypothalamus associated with decreased hypothalamic ER stress [210]. These phenotypes were recapitulated by both T3 administration and the expression of a dominant-negative version of AMPK in the ventromedial hypothalamus [210]. Therefore, the authors proposed a model whereby AMPK activity is inhibited by thyroid hormone action to suppress ceramide production and ER stress in the hypothalamus, suggesting a role of AMPK in regulating ceramide levels in cellular stress and metabolic control [210]. Since AMPK activity is stimulated within the AdipoR signaling cascade, additional ceramidase-independent effects of AdipoRs on ceramide metabolism could be related to adiponectin-stimulated changes in AMPK activity. Together, these findings support the notion that the metabolic actions of AMPK are partly mediated by reducing cellular ceramide levels. As a result, decreased AMPK action in obesity may have causal roles in ceramide accumulation, thereby decreasing insulin sensitivity in multiple organs.

### Endocannabinoids

Endocannabinoids are endogenous lipid-based retrograde neurotransmitters that act via cannabinoid receptors, including CB_1_R, expressed in the central nervous system and peripheral tissues to regulate various metabolic processes [211, 212]. In obesity, the endocannabinoid system is often highly active, while its ablation or inhibition reduces body weight and improves insulin sensitivity [213–215]. Studies in human glioma cells have revealed that cannabinoid action triggers ceramide accumulation either acutely through sphingomyelin hydrolysis or sustainedly through de novo synthesis via regulation of SPT activity [216]. In diet-induced obese mice, blockage of CB_1_R by chronic treatment with a peripherally restricted inverse agonist (JD5037) attenuated the diet-induced increases in hepatic C_14:0_, C_16:0_, C_18:0_, and C_20:0_ ceramides and improved glucose tolerance and insulin sensitivity [217]. From a mechanistic point of view, CB_1_R inverse agonism reversed the high-fat diet-dependent increase in SPT activity, decreased the expression of ceramide biosynthetic genes (*Sptlc3*, *CerS1*, *CerS6*), and increased the expression of ceramidases (*Asah1*, *Asah2*) [217]. These observations have led the authors to conclude that the ceramide-lowering effects and beneficial metabolic outcomes of CB_1_R inhibition are due to both reduced ceramide de novo synthesis and increased ceramide degradation [217]. Accordingly, it is tempting to speculate that increased CB_1_R signaling during obesity development contributes to tissue ceramide accumulation.

### Intestinal transcription factors

In the intestine, specific transcriptional regulators have been associated with ceramide production and secretion in the pathophysiology of obesity-related metabolic pathologies [218]. Convincing evidence is provided by a series of studies from the Gonzalez group showing that the intestinal farnesoid X receptor (FXR) promotes ceramide synthesis in the gut, leading to systemic increases in ceramide content that can trigger liver steatosis and systemic metabolic defects [219–221]. In turn, inhibition of intestinal FXR in obese mice decreases ceramide levels both in the intestine and circulation, which resolves hepatic steatosis and enhances the thermogenic capacity of adipose tissue, in part through increased mitochondrial uncoupling and adipose tissue browning to ameliorate obesity and insulin resistance [219–221]. Neutral sphingomyelinase (*Smpd3*), encoding for nSMase2, was recently identified as an FXR target gene mediating the effects on intestinal ceramide (mainly C_16:0_) production and secretion, also in the pathophysiology of atherosclerosis [222]. The gut microbiota has been implicated as an environmental factor that modulates obesity and its related diseases through FXR [223]. Ceramides may thus be critical determinants of a subject’s susceptibility to developing metabolic diseases in obesity related to specific alterations of the intestinal microbiome.

In addition, intestinal ceramide levels appear to be under the control of HIF2α, a transcription factor stabilized under hypoxic conditions [224]. HIF2α was found to govern transcriptional control over neuraminidase 3 (*Neu*3), encoding a key enzyme in the ceramide salvage pathway [224]. In this study, disruption of intestinal HIF2α in mice reduced intestinal and circulating ceramide levels during high-fat diet feeding (most notably C_16:0_ ceramides), accompanied by reductions in body weight gain and hepatic steatosis, and improvements in systemic insulin sensitivity [224]. HIF2α-dependent effects on ceramide turnover also occur in hypoxic WAT, but through a distinct mechanism that involves transcriptional regulation of alkaline CDase (*Acer2*), and this process has been linked to the pathophysiology of atherosclerosis [225].

It was recently found that the transcriptional regulator MYC also interferes with ceramide production, thus modulating intestinal and systemic ceramide levels in obesity [226]. Similar to *Fxr* and *Hif2a*, *Myc* expression in the intestine is increased in obesity [226]. In turn, disruption of *Myc* in intestinal epithelial cells led to a reduction in serum ceramide levels in mice and ameliorated HFD-induced obesity and hepatic steatosis [226]. The changes in ceramide content following MYC disruption were attributed to changes in the expression of *CerS4*, which turned out to be a MYC target gene increased in the intestine of obese subjects [226]. However, proof of a casual relationship between intestinal CerS4-dependent ceramide synthesis and obesity-related metabolic diseases is pending. Nevertheless, the studies collectively indicate that altered regulation of specific transcription factors in the intestine affects endogenous ceramide production. Although FXR, HIF2*a*, and MYC alter ceramide formation through unique processes, each system promotes the delivery of ceramides from the intestine to other tissues, including the liver, thereby impairing systemic metabolic integrity in obesity.

### Other factors

Additional pathways that may contribute to ceramide accumulation in obesity by modulating ceramide metabolic rate are currently discussed. For example, β-adrenergic signaling was found to efficiently shut down ceramide synthesis in primary adipocytes [25]. However, the molecular targets downstream of the β-adrenergic receptor involved in this process and its implication in the obesity-related accrual of ceramides in adipose tissue are still unclear. In addition, studies in yeast point toward a role of the Target of Rapamycin (TOR) complex 2 (TORC2), which is closely related to obesity and metabolic control, in regulating SPT-dependent ceramide formation and CerS phosphorylation [227, 228].

The mechanisms by which the pathways presented here modulate ceramide content in obesity often remain vaguely defined, and the vast majority of studies report correlative changes in the expression of proteins involved in general ceramide turnover, such as SPT, DES, or CDase, regardless of whether their alteration is the cause or consequence of altered metabolic control. In addition, it is assumed that these enzymes do not have substrate specificity or preference for certain acyl chain length molecular species and can thus be attributed to the obesity-related changes in specific ceramides only upon differential availability of precursor substrates driving selective ceramide synthesis. At the same time, altered regulation of CerSs is thought to promote the obesity-related increases in acyl chain length-specific de novo ceramide formation. However, the CerS-modifying signaling pathways involved in this process remain largely unknown. Indeed, there is a vast opportunity embedded in understanding how CerS activity is regulated in obesity (e.g., at the transcriptional and post-translational level) to change the content of specific ceramide species and influence systemic glucose homeostasis, and this needs to be an intensive area of investigation in the near future.

## Relevant tissues of ceramide metabolism and action in obesity

**Fig. 4:**
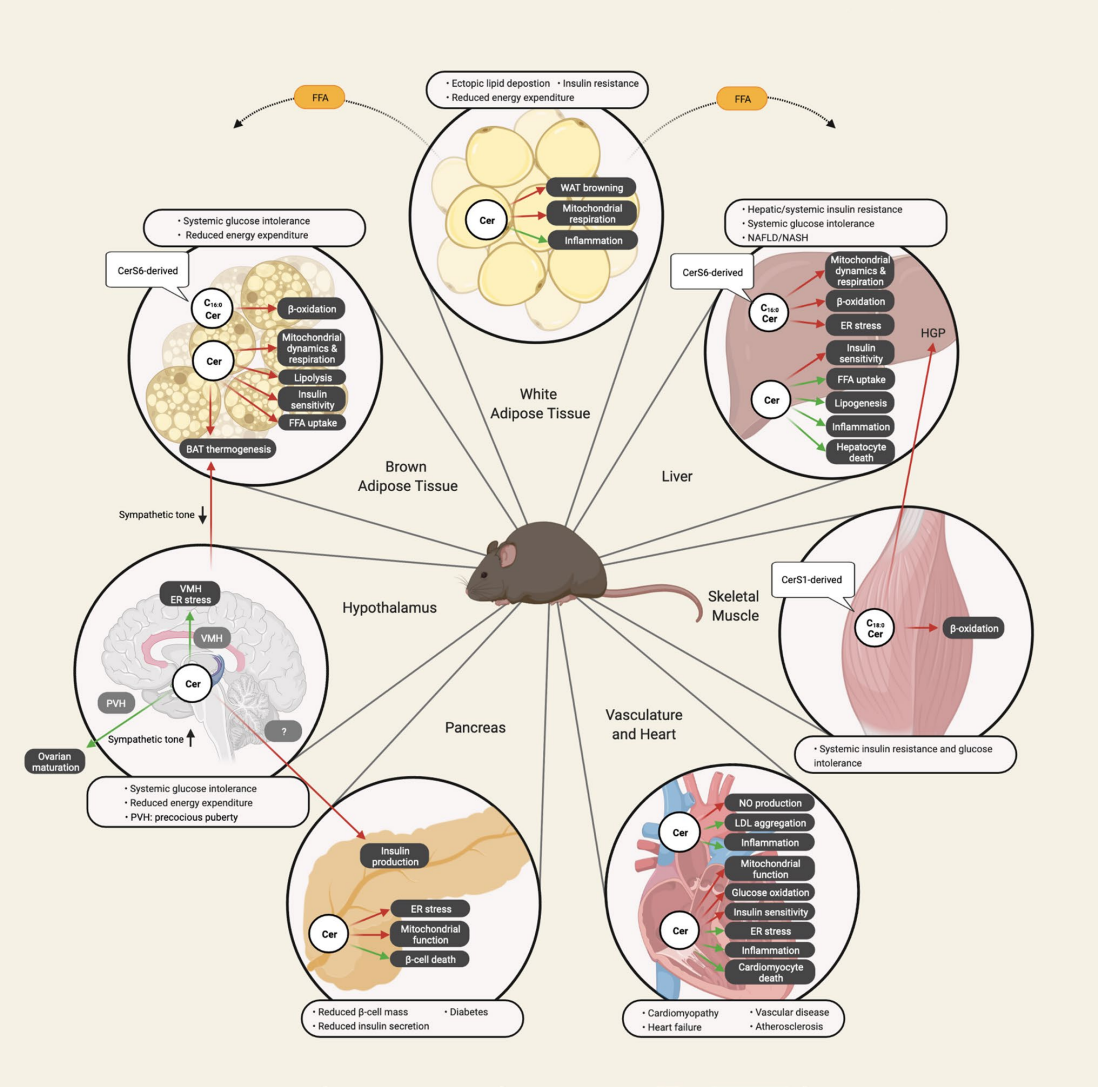
Tissue-specific effects of ceramide accumulation and the related health consequences in obesity. Most conclusive observations have been demonstrated in rodent models of obesity or dyslipidemia. Although ceramides have been associated with obesity-related metabolic dysfunction and disease development in all tissues shown, the exact ceramide molecular species involved in these processes often remain undefined. If there is evidence of the ceramide species promoting tissue-specific lipotoxicity, this is indicated accordingly. Red arrows indicate inhibitory effects, and green arrows indicate stimulatory effects. *Cer* ceramide, *CerS* ceramide synthase, *ER* endoplasmic reticulum, *FFA* free fatty acid, *HGP* hepatic glucose production, *LDL* low density lipoprotein, *NAFLD* non-alcoholic fatty liver disease, *NASH* non-alcoholic steatohepatitis, *NO* nitric oxide, *PVH* paraventricular hypothalamus, *VMH* ventromedial hypothalamus

Ceramide accumulation and the associated metabolic effects in obesity are highly organ-specific (Fig. 4). This has been demonstrated by the use of model organisms together with (sphingo)lipidomic analyses in human tissue biopsies and rodents with obesity and/or dyslipidemia. Inhibition or overexpression of specific CerSs in murine models has started to provide evidence about the molecular nature of the specific ceramide species eliciting lipotoxic responses in obesity and further demonstrated that inhibiting chain length-specific ceramide synthesis in individual tissues can substantially improve metabolic homeostasis. In this context, challenges in interpreting data obtained from sphingolipidomic analyses and CerS interference should be noted, as we discuss in (Box 2). Below, we present a selection of key findings that have contributed to our current understanding of the tissue-specific regulation and functional roles of ceramides in the pathophysiology of obesity-related metabolic diseases.

### Ceramides in the adipose tissue

White adipose tissue (WAT) is a multifactorial organ that can store large amounts of TAGs and communicate the status of endogenous fat storage to other tissues by endocrine signaling to adapt nutrient intake, storage, and utilization [229]. These processes are often disturbed in obese individuals triggering dyslipidemia and metabolic dysfunction [229]. While expanding visceral WAT depots are associated with risk for metabolic disease, anatomically distinct depots of subcutaneous WAT may elicit more protective effects on energy homeostasis [229].

Human correlative studies have demonstrated an association between increased ceramide content in distinct adipose tissue depots and obesity-related pathologies [23, 230–234]. For example, C_16:0_ ceramides were elevated in subcutaneous- but not in visceral WAT in a small cohort of obese patients with type 2 diabetes as compared to obese non-diabetics [23]. In a small group of obese non-diabetic women with hepatic steatosis, C_24:1_ ceramides showed increased levels in the inflamed subcutaneous WAT [231]. In another group of obese women, higher total ceramide levels were measured in visceral- compared to subcutaneous WAT, where C_16:0_ and C_18:0_ ceramides related to systemic metabolic defects [233]. Moreover, in a study involving subjects across different BMIs, an increase in most ceramides was recorded in both the subcutaneous- and visceral epicardial WAT depot in obese individuals, with a close association between C_16:0_ ceramides in subcutaneous WAT and high HOMA-IR [232]. A challenging task will be to understand the variability of obesity-related changes in ceramide content within the different WAT depots between groups of patients.

Recently, an in-depth lipidomic profile of human WAT (AdipoAtlas) confirmed that C_16:0_ ceramides with the usual sphingoid base sphingosine (d18:1) are the most abundant species also in the adipose tissue of humans [234]. In addition, the authors found that WAT exhibits high relative amounts of potentially lipotoxic deoxy-ceramides (> 10% of all ceramide subclasses), a yet poorly studied ceramide molecular species produced from alanine instead of serine [234]. The obese WAT showed a marked upregulation of ceramides with the unusual sphingoid base sphingadienine (d18:2) with a variety of amide-linked acyl chains (C_14:0_-C_24:0_), illustrating the need for analysis of ceramides also with alternative sphingoid bases in the context of obesity-related WAT dysfunction [234].

In genome-wide association studies, the SPT suppressor *ORMDL3* was identified as an obesity-related gene, and its expression in human subcutaneous WAT inversely correlates with BMI [235, 236]. Conversely, *Ormdl3*-deficient mice show elevated ceramide levels in WAT, increased body weight gain upon high-fat diet feeding, and insulin resistance, which was attributed to decreased thermogenesis and impaired WAT browning [235]. Myriocin treatment reversed the detrimental phenotypes in these mice, indicating that the inhibition of ceramide synthesis by ORMDL3 is a critical mechanism for maintaining adipose tissue function and systemic energy homeostasis [235]. Furthermore, by adipocyte-specific deletion of *Sptlc2* in obese mice, Chaurasia and colleagues found that the inhibition of ceramide synthesis decreased C_24:0_ and C_24:1_ ceramides in epididymal- and C_16:0_–C_24:0_ and C_24:1_ ceramides in subcutaneous WAT, which improved adipose tissue function preferentially in subcutaneous depots [23]. More specifically, adipocyte-specific *Sptlc2* deficiency stimulated M2 macrophage polarization, increased thermogenic gene expression, and abolished inflammation [23]. These animals showed improved energy expenditure, insulin sensitivity, glucose tolerance, and decreased hepatic steatosis, indicating that the adipocyte-autonomous effects of ceramides affect systemic metabolic function [23]. Similarly, adipocyte-specific deletion of *Degs1* in obese mice improved systemic insulin sensitivity and glucose tolerance but without effects on adiposity and energy expenditure [25]. Beneficial effects were also observed in high-fat diet-fed mice with inducible adipocyte-specific overexpression of CDase, which reduced total ceramide levels in different visceral and subcutaneous depots (C_16:0_ and C_18:0_ ceramides showed the most consistent and robust decrease (± 50%)) that markedly improved systemic and adipose tissue-specific insulin sensitivity [44]. Adipocyte-specific CDase overexpression in obesity also reduced C_16:0_ and C_18:0_ ceramides in the liver, associated with improved hepatic insulin sensitivity and protection from diet-induced hepatic steatosis [44]. In contrast to most studies showing that ceramide-lowering interventions in adipose tissue alleviate metabolic dysfunction in obesity, it was reported that reducing ceramide content by the deletion of *Sptlc1* or *-2* impairs adipose tissue remodeling and causes lipodystrophy, which may have occurred due to impairments in adipocyte differentiation [237, 238]. These findings reemphasize the need to identify and selectively modulate the specific lipotoxic ceramide species in adipose tissue to avoid the adverse effects associated with reducing other ceramides crucial for maintaining adipocyte function and survival.

C_16:0_ ceramides were investigated in more detail concerning their roles in regulating adipose tissue function in obesity. An increase in the content of C_16:0_ ceramides in both visceral and subcutaneous WAT can be observed in mice and humans and may be attributed to increased CerS6-dependent ceramide synthesis in these tissues [27]. In a cohort of 439 obese versus lean subjects, significant correlations were found between WAT *CERS6* mRNA expression and BMI, systemic insulin resistance, adipocyte size, and circulating leptin and HbA1c levels [27]. Conversely, the specific blockage of C_16:0_ ceramide production through conventional knockout of *CerS6* prevented the diet-induced elevations in C_16:0_ ceramides in WAT, which led to decreased body fat content, reduced adipocyte size, and reduced macrophage WAT infiltration [27]. These findings have indicated that the accumulation of CerS6-derived C_16:0_ ceramide in WAT is involved in the obesity-related impairment of WAT function; however, an adipocyte-specific model of CerS6 deficiency has not yet been described, which is necessary to conclude adipocyte-autonomous effects of CerS6-derived C_16:0_ ceramides in vivo.

Distinct from WAT are depots of BAT with high mitochondrial density that dissipate energy to produce heat. In BAT of obese rodents, de novo ceramide synthesis (particularly of C_16:0_ and C_18:0_ ceramides) is increased [24, 27]. Conversely, decreased ceramide synthesis was found in primary brown adipocytes following β-adrenergic stimulation due to reduced expression of *Sptlc2* and *CerS6* [24]. In rodents, β-adrenergic agonists are effective thermogenic, anti-obesity, and insulin-sensitizing agents that exert their effects primarily through actions in WAT, BAT, and muscle. Thus, it is postulated that the metabolically beneficial effects of β-adrenergic stimulation are partly due to the blockage of ceramide build-up in these tissues. Accordingly, deletion of *Sptlc2* in UCP1-positive brown adipocytes protects mice from diet-induced obesity by increasing BAT function and systemic energy expenditure [24]. In turn, thermogenic regulation is impaired when ceramide degradation is inhibited through BAT-specific deletion of acid CDase (*Asah1*), exacerbating obesity, hepatic steatosis, and insulin resistance [24]. Interestingly, these phenotypes were related to changes in mitochondrial structure and function, respectively [24]. Whereas the inhibition of ceramide synthesis increased mitochondrial density, size, and respiration in BAT of obese mice, blocking ceramide degradation in lean animals decreased mitochondrial density and impaired mitochondrial respiration [24]. Thus, ceramides may be involved in the deregulation of mitochondrial morphology and respiratory function in BAT with adverse metabolic consequences. Specifically, increased formation of CerS6-derived C_16:0_ ceramides in primary brown adipocytes is sufficient to disturb mitochondrial morphology and function [24], and the deletion of *CerS6* in UCP1-positive cells increases mitochondrial β-oxidation capacity in BAT to improve energy expenditure and glucose tolerance in diet-induced obese mice [27]. Together, these studies support the notion that CerS6-derived C_16:0_ ceramides promote metabolic dysfunction at least in part through adverse actions on mitochondrial function in brown adipocytes. C_16:0_ ceramides may also impair brown adipogenesis, thus contributing to impairments of the aged BAT [239]. Chaurasia and colleagues made additional suggestions that ceramides in BAT slow lipolysis by inhibiting HSL phosphorylation, inhibit insulin-stimulated AKT phosphorylation, and reduce FFA uptake [24].

### Ceramides in the liver

Excessive hepatic lipid storage in obesity can lead to non-alcoholic fatty liver disease (NAFLD) and steatohepatitis (NASH). These conditions are characterized by a particular increase in the liver TAG content, often associated with hepatic and systemic insulin resistance (NAFLD) alongside inflammation that can promote hepatic cirrhosis and fibrosis (NASH) [240]. TAG concentrations in the liver exhibit a strong predictive value for insulin resistance [241], which is believed to arise from the secondary accumulation of deleterious TAG metabolites, including ceramides. Hepatic ceramide levels increase in conditions of dyslipidemia and insulin resistance, e.g., in the liver of lard oil- or dexamethasone-infused rats, high-fat diet-fed mice, and genetically obese rodent models [12, 81, 192]. This is attributable to increases in specific ceramide species (most consistently C_16:0_–C_20:0_), which in rodents positively correlate with the degree of steatosis, insulin resistance, hepatic oxidative stress, and inflammation, while other ceramide species remain unaltered or even decrease under these conditions [26, 27, 33, 217, 242–249] (Table 1). Consistent with the data obtained in rodents, specific ceramide species are elevated in the liver of humans with NAFLD. Luukkonen and colleagues observed that hepatic C_16:0_ and C_18:0_ ceramides derived from de novo synthesis in obese patients with NAFLD strongly correlate with insulin resistance as measured by HOMA-IR [250]. Intriguingly, patients with NAFLD induced by a mutation in the gene encoding patatin-like phospholipase domain-containing protein 3 (PNPLA3), who are relatively protected against insulin resistance, did not show the same increase in hepatic ceramide levels, linking ceramide accumulation in NAFLD to the development of metabolic deficits [250]. Similarly, an increase in total hepatic ceramide content and that of specific dihydroceramide species (C_16:0_, C_22:0_, C_24:1_) was found in patients with NASH compared to those having fatty liver disease without hepatic inflammation [251].

**Table 1:** Examples of experimental in vivo evidence that specific ceramide species increase in the liver of obese rodents and cause metabolic deterioration

Author (Year)	Model	Genotype (Strain) + Intervention	Diet	Hepatic ceramides	Metabolic phenotype	References
Bikman et al. (2012)	Mouse	wild-type (C57Bl/6N)	CD vs HFD (60% cal fat), 12w	C_16:0_↑, C_20:0_↑, C_22:0_↑	• Obesity • Insulin resistance and glucose intolerance	[33]
		wild-type + Fenretinide	HFD	C_16:0_↓, C_20:0_↓, C_22:0_↓	• Improved glucose tolerance and insulin sensitivity	
Cinar et al (2013)	Mouse	wild-type (C57Bl/6J)	CD vs HFD (60% cal fat), 16w	C_14:0_↑, C_16:0_↑, C_18:0_↑, C_20:0_↑, C_24:0_↓	• Insulin resistance and glucose intolerance • Impaired hepatic insulin sensitivity	[217]
		wild-type + Myriocin	HFD	C_14:0_↓, C_16:0_↓, C_18:0_↓, C_20:0_↓, C_24:0_↓	• Improved glucose tolerance and insulin sensitivity • Improved hepatic insulin sensitivity	
		wild-type + JD5037 (CB_1_R antagonist)	HFD	C_14:0_↓, C_16:0_↓, C_18:0_↓, C_20:0_↓, C_24:0_↑	• Improved glucose tolerance and insulin sensitivity • Improved hepatic insulin sensitivity	
Turpin et al. (2014)	Mouse	wild-type (C57Bl/6N)	CD (12% cal fat) vs HFD (55.2% cal fat), 17w	C_14:0_↑, C_16:0_↑, C_18:0_↑, C_20:0_↑, C_24:1_↑	• Obesity	[27]
		*CerS6* ^whole-body^ ^KO^ vs CerS6 ^WT^	HFD	C_16:0_↓	• Protection from DIO • Protection from glucose intolerance • Increased mitochondrial β-oxidation	
		*CerS6* ^Liver KO^ vs CerS6^fl/fl^	HFD	C_16:0_↓	• Improved glucose tolerance • Improved hepatic insulin sensitivity	
Kasumov et al. (2015)	Mouse	LDLR^−/−^	CD (10% cal fat) vs HFD (45% cal fat), 12w	C_16:0_↑, C_18:0_↑, C_20:0_↑, C_24:0_↓	• Obesity • Hepatic oxidative stress, inflammation, apoptosis • Insulin resistance and glucose intolerance	[244]
		LDLR^−/−^ + Myriocin	HFD	C_16:0_↓, C_18:0_↓, C_20:0_↓, C_22:0_↓, C_24:1_↓	• Improved systemic glucose tolerance and hepatic insulin sensitivity • Decreased hepatic steatosis, apoptosis, fibrosis	
Zabielski et al. (2018, 2019)	Wistar rat	wild-type	CD (10% cal fat) vs HFD (60% cal fat), 8w	C_14:0_↑, C_16:0_↑, C_18:0_↑, C_20:0_↑, C_22:0_↑, C_24:0_↑	• Insulin resistance and glucose intolerance	[245, 246]
		wild-type + Metformin	HFD	C_14:0_↓, C_16:0_↓, C_18:0_↓, C_20:0_↓, C_22:0_↓, C_24:0_↓, C_24:1_↓	• Improved glucose tolerance and insulin sensitivity	
		wild-type + Myriocin	HFD	C_14:0_↓, C_16:0_↓, C_18:0_↓, C_20:0_↓, C_22:0_↓, C_24:0_↓, C_24:1_↓	• Improved glucose tolerance and insulin sensitivity	
Raichur et al. (2019)	Mouse	ob/ob vs lean	CD	C_16:0_↑, C_18:0_↑, C_20:0_↑, C_22:0_↓, C_24:0_↓	• Obesity • Insulin resistance and glucose intolerance	[243]
		ob/ob + *CerS6* antisense-oigonucleotide (ASO)	CD	C_16:0_↓	• Reduced weight gain • Improved glucose tolerance and insulin sensitivity	
Hammerschmidt et al. (2019)	Mouse	wild-type (C57Bl/6N)	CD (13% cal fat) vs HFD (60% cal fat), 16w	Whole liver: C_14:0_↑, C_16:0_↑, C_18:0_↑, C_20:0_↑, C_22:1_↑, C_24:0_↑	• Obesity • Hepatic mitochondrial fragmentation	[26]
				Mitochondria: C_16:0_↑, C_18:0_↑		
		*CerS6* ^whole-body^ ^KO^ vs *CerS6* ^WT^	HFD	Whole liver: C_14:0_↓, C_16:0_↓, C_18:1_↓, C_22:1_↓	• Protection from DIO and hepatic steatosis • Protection from hepatic mitochondrial fragmentation • Improved hepatic mitochondrial respiration • Protection from glucose/pyruvate intolerance and insulin resistance	
				Mitochondria: C_14:0_↓, C_16:0_↓, C_18:0_↓, C_18:1_↓, C_20:0_↓, C_22:1_↓		
		*CerS5* ^whole-body^ ^KO^ vs *CerS5* ^WT^	HFD	Whole liver: C_16:0_↓	• Obesity (similar to controls) • Hepatic mitochondrial fragmentation (similar to controls) • Glucose intolerance and insulin resistance (similar to controls)	
				Mitochondria: C_20:0_↑, C_22:0_↑, C_22:1_↑, C_24:0_↑, C_24:1_↑		
		*CerS6* ^inducible peripheral KO^ vs *CerS6*^fl/fl^	HFD	C_16:0_↓	• Improved glucose/pyruvate tolerance and insulin sensitivity • Restoration of hepatic mitochondrial morphology	
		*CerS6*^fl/fl^ + AAV8-TBG-CRE vs AAV8-TBG-GFP	HFD	C_16:0_↓	• Improved glucose tolerance • Trend toward improved insulin sensitivity and pyruvate tolerance	
		wild-type + AAV8-TBG-CerS6 vs AAV8-TBG-GFP	CD	C_16:0_↑	• Hepatic Mitochondrial fragmentation • Mild impairment in glucose metabolism	

For the representation of this table, only studies were chosen that quantified different ceramide species in obesity/NAFLD and additionally presented interventions to reduce ceramides

Evidence for a causal role of ceramides in the liver to systemic metabolic dysfunction is given from obese animal models in which disruption of ceramide synthesis consistently prevented or reversed hepatic ceramide accumulation and improved insulin sensitivity, glucose tolerance, and hepatic steatosis [12, 29, 33, 217, 244–246, 252, 253]. Liver-specific effects of ceramides were demonstrated through disruption of ceramide synthesis in obese mice (via hepatocyte-restricted deletion of *Degs1*) or stimulation of ceramide degradation (via hepatocyte-restricted stimulation of CDase expression or activity), which sufficiently attenuated hepatic steatosis and improved systemic glucose metabolism [25, 44, 192, 203, 205]. These studies have indicated that the liver presents a key site for the adverse effects of ceramide accumulation in obesity and that interventions to reduce hepatic ceramides are promising to alleviate obesity-related metabolic defects. Specifically, of the many ceramide species, CerS6-derived C_16:0_ ceramides elicit lipotoxic reactions in the liver [26, 27, 93, 243] (Table 1). Both mRNA and protein expression of CerS6 is increased in the liver of diet-induced obese mice concomitant with increased hepatic C_16:0_ ceramide content [26]. Conversely, improved glucose metabolism in high-fat diet-fed or genetically obese mice can be achieved by conventional [27], whole-body inducible [26, 243], and liver-specific disruption of CerS6-dependent C_16:0_ ceramide synthesis. Beneficial effects of reducing C_16:0_ ceramides were also observed in mice fed a methionine-choline deficient diet that promotes NASH independent of obesity [254]. In these animals, treatment with the GLP-1 receptor agonist liraglutide prevented the diet-induced accumulation of C_16:0_ and C_24:0_ ceramides in the liver and alleviated hepatic inflammation and fibrosis [254]. These findings have demonstrated the multifaceted effects on liver physiology induced by C_16:0_ ceramide accumulation. Other studies have indicated that specific ceramides (C_16:0_ and C_18:0_) accumulate during the progression from the steatotic liver to the NASH liver in mice fed a fat- and cholesterol-rich diet [255]. In turn, diminishing hepatic ceramide synthesis by Myriocin treatment can prevent steatosis and fibrosis by ameliorating hepatic inflammation, autophagy, and apoptosis in high-fat diet-induced obese rats [256, 257].

Substantial differences exist between CerS5- and CerS6-derived C_16:0_ ceramides in the liver, as only reducing the latter in mice prevents the development of diet-induced insulin resistance and hepatic steatosis [26]. We found that the deletion of CerS6 in mice results in reduced C_16:0_ ceramide levels in mitochondria and MAM [26]. This protected the animals against diet-induced mitochondrial fragmentation in hepatocytes and improved mitochondrial respiratory function, highlighting the significance of the subcellular site of ceramide accumulation [26]. Furthermore, liver-specific targeting of CerS6 to reduce hepatic C_16:0_ ceramide synthesis in obesity can even reverse the detrimental effects of obesity on mitochondrial morphology and glucose metabolism, highlighting the therapeutic potential of CerS6 inhibition to treat obesity-associated hepatic and systemic defects [26]. CerS6-derived C_16:0_ ceramides in the liver also promote ER stress in the modulation of lipogenesis and hepatic lipid loading upon fatty acid excess by stimulating SREBP1 processing [95]. The ceramide-dependent regulation of CD36-dependent fatty acid uptake in hepatocytes may be relevant in this process [25, 27, 44]. Collectively, these observations indicate that C_16:0_ ceramides, as generated by CerS6 in hepatocytes, elicit diverse effects on liver metabolism in the obesity-associated deterioration of liver-specific and systemic metabolic homeostasis.

While the roles of hepatic ceramides in promoting metabolic disease have been a significant focus, some evidence points toward beneficial effects of specific ceramide species in this context. In mouse models of type 1 diabetes and diet-induced obesity, C_24:1_ ceramides were reduced in the liver and plasma [258]. In turn, restoring hepatic C_24:1_ ceramides by dietary supplementation of nervonic acid, a C_24:1_ ω-9 fatty acid, reduced body weight gain and improved glucose tolerance and insulin sensitivity [259]. Similarly, elevated C_18:1_ ceramides in mice fed a palmitate-enriched diet by deleting alkaline CDase 3 (*Acer3*) alleviated early inflammation and fibrosis, possibly by suppressing hepatocellular oxidative stress in the NASH liver [260]. These results underscore the distinct and partly opposing roles of different ceramide species and point out the need to consider differential changes in the array of hepatic ceramides concerning metabolic disease development and progression.

### Ceramides in the skeletal muscle

Skeletal muscle is a highly active metabolic organ and a key site for glucose disposal during hyperglycemia depending on insulin action in myocytes. In obesity, skeletal muscle accumulates lipids caused by an imbalance of nutrient supply and utilization, with lipotoxic effects on insulin sensitivity. This phenomenon used to be under controversial debate given the “athlete’s paradox” that arose from observations in endurance-trained athletes, which exhibit enhanced muscle insulin sensitivity but high amounts of intramyocellular glycerolipids (particularly TAGs) [261]. This phenomenon indicates that the classic neutral lipids are unlikely to be the primary cause of insulin resistance in muscle, which has switched the focus to other lipid metabolites. Studies have recently demonstrated a more consistent link between skeletal muscle ceramide content and impaired insulin sensitivity in rodent models and humans [262].

In skeletal muscle, C_18:0_ ceramides account for most ceramide content, which in mice almost entirely depends on muscle-specific CerS1-mediated ceramide formation [28]. Endogenous ceramide production in skeletal muscle is increased in mouse models of diet-induced obesity, which show increased CerS1 expression and elevated muscle C_18:0_ ceramide content [28, 263]. This can be observed in mice as early as three weeks of high-fat diet feeding, accompanied by reduced glucose tolerance [264]. The Watt laboratory has identified an additional mechanism of ceramide accrual in muscle in type 2 diabetic patients, involving LDL-mediated transport of ceramides and uptake by myotubes, which is sufficient to induce systemic insulin resistance by mediating decreased insulin action in muscle [265]. While skeletal muscle C_18:0_ ceramides are consistently increased in obese rodent models (Table 2), other ceramide species (C_14:0_, C_24:0_, and C_24:1_) decrease after chronic HFD feeding in rats, pointing towards the differential roles of selected ceramide species in muscle [266]. Similarly, most lipid profiling studies on human muscle biopsies revealed increased ceramide content in obesity and diabetes [10, 267–270], with a C_18:0_ ceramide signature for insulin resistance [271, 272]. In particular, increased C_18:0_ ceramide content was found in the muscle of obese and insulin-resistant individuals compared to obese and insulin-sensitive or lean counterparts [271]. Similarly, C_18:0_ ceramides were increased in skeletal muscle of obese subjects with type 2 diabetes compared to obese non-diabetics and decreased in endurance-trained athletes, especially after acute exercise [272]. Correlation analysis further revealed a strong association between skeletal muscle C_18:0_ ceramides and BMI and an inverse relationship with insulin sensitivity [272]. In turn, weight loss interventions in humans, such as acute [273] and chronic exercise training [267, 274–276], or bariatric surgery [277], consistently decrease muscle ceramide content in conjunction with improved insulin sensitivity and, in some cases, increased skeletal muscle β-oxidation. However, no such correlations were found in a minor subset of lipid profiling studies, which has sparked a controversial debate on the extent to which muscle ceramides were involved in regulating insulin sensitivity at all [278, 279]. However, these conflicting results reemphasize that it may not be the total content of muscle ceramides that matters but changes in specific ceramide species in discrete subcellular pools. In support of this notion, a correlation study on human muscle biopsies suggested that the accumulation of C_18:0_ ceramides, specifically in a mitochondrial/ER subsarcolemmal fraction, underlies decreased insulin sensitivity [109].

Data obtained from in vitro experiments and murine models confirm that ceramides in myocytes play a crucial role in the deterioration of systemic glucose metabolism. Thus, inhibition of ceramide synthesis in cultured myotubes prevents palmitate-induced changes in mitochondrial morphology and function and restores insulin sensitivity [83, 118]. Similarly, reducing ceramide content in muscle of obese rodents, e.g., by blocking general ceramide synthesis using Myriocin treatment, consistently improves energy expenditure and glucose homeostasis [12, 30, 118, 263, 266] (Table 2). Knockout experiments in mice have further confirmed the exceptionally critical role of CerS1-derived C_18:0_ ceramides in skeletal muscle in obesity [28]. Ablation of muscle C_18:0_ ceramide synthesis by myocyte-specific knockout of CerS1 significantly improved insulin sensitivity in high-fat diet-fed mice despite unchanged adiposity [28]. In contrast, the myocyte-specific deletion of CerS5 and CerS6 did not affect insulin sensitivity and glucose metabolism in obesity, highlighting the tissue-specific regulation and roles of distinct CerSs and their specific ceramide products [28]. Surprisingly, deleting CerS1 in skeletal muscle to reduce C_18:0_ ceramide synthesis in obesity did not appreciably affect muscle-specific insulin signaling or glucose uptake [28]. Still, it improved insulin’s ability to suppress hepatic glucose production and systemic glucose tolerance through increased circulating concentrations of muscle-derived FGF21 [28]. FGF21 is a proteotypic effector of an integrated stress response, particularly to mitochondrial damage [280]. This finding might indicate that CerS1 regulates mitochondrial integrity in vivo, but no overt structural or functional changes of mitochondria were observed in skeletal muscle due to myocyte-specific CerS1 deficiency [28]. Interestingly, pharmacologic inhibition of CerS1 in diet-induced obese mice with a recently developed specific inhibitor (P053) demonstrated a role of CerS1 inhibition on muscle fatty acid oxidation [34]. P053 treatment in high-fat diet-fed mice decreased muscle C_18:0_ ceramide content by half, associated with increased mitochondrial β-oxidation and significantly reduced TAG levels in muscle [34]. However, compared to the more comprehensive genetic inactivation of CerS1, partial pharmacological inhibition of CerS1 did not alter systemic insulin sensitivity or glucose tolerance [34]. Still, it is predicted that CerS1-derived C_18:0_ ceramides cause metabolic deterioration in obesity and that CerS1 inhibition provides a promising strategy for treating obesity-related metabolic abnormalities.

**Table 2:** Examples of experimental in vivo evidence that specific ceramide species increase in the skeletal muscle of obese rodents and cause metabolic deterioration

Author (Year)	Model	Genotype + Intervention	Diet	Skeletal muscle ceramides	Metabolic phenotype	References
Bikman et al. (2012)	Mouse	wild-type	CD vs HFD (60% cal fat), 12w	C_16:0_↑, C_18:0_↑	• Obesity • Insulin resistance and glucose intolerance	[33]
		wild-type + Fenretinide	HFD	C_16:0_↓, C_18:0_↓	• Improved glucose tolerance and insulin sensitivity	
Blachnio-Zabielska et al. (2016)	Rat	wild-type	CD (10% cal fat) vs HFD (60% cal fat), 8w	C_14:0_↓, C_18:0_↑, C_18:1_↑, C_24:0_↓, C_24:1_↓	• Insulin resistance and glucose intolerance • Impaired muscle insulin sensitivity	[266]
		wild-type + Myriocin	HFD	C_14:0_↑, C_16:0_↓, C_18:0_↓, C_18:1_↓, C_20:0_↓	• Protection from glucose intolerance and insulin resistance • Improved muscle insulin sensitivity	
Turner et al. (2018)	Mouse	wild-type	CD (8% cal fat) HFD (45% cal fat), 4-5w	C_24:1_↓	• Obesity • Insulin resistance, hyperinsulinemia, and glucose intolerance	[34]
		wild-type + PO53	HFD	C_18:0_↓, C_22:0_↑, C_24:0_↑, C_24:1_↑	• Protection from obesity • Increased fatty acid β-oxidation and mitochondrial respiration	
Turpin-Nolan et al. (2019)	Mouse	wild-type	CD (12% cal fat) vs HFD (55.2% cal fat), 24w	C_14:0_↓, C_18:0_↑, C_26:0_↓	• ND	[28]
		CerS1 ^whole-body^ ^KO^ vs CerS1 ^WT^	HFD	C_16:0_↑, C_18:0_↓, C_22:0_↑, C_22:1_↑, C_24:0_↑, C_24:1_↑	• Protection from obesity • Protection from glucose intolerance and insulin resistance • Increased energy expenditure	
		CerS1 ^SkM^ ^KO^ vs CerS1 ^fl/fl^	HFD	C_18:0_↓, C_18:1_↓, C_22:0_↓, C_22:1_↑, C_24:0_↑, C_24:1_↑	• Protection from glucose intolerance and insulin resistance	

For the representation of this table, only studies were chosen that quantified different ceramide species in obesity and additionally presented interventions to reduce ceramides

### Ceramides in the pancreas

Pancreatic β-cells located in the islets of Langerhans are integral to the systemic control of glucose homeostasis through their unique ability to produce and secrete insulin. Evidence suggests that high concentrations of fatty acids and glucose in obesity trigger glucolipotoxic responses that gradually impair the ability of β-cell to provide insulin due to dedifferentiation or altered mass, which is a hallmark of late-stage type 2 diabetes mellitus [281, 282]. Accordingly, mitigation of plasma FFA levels prevents β-cell dysfunction in obese rodents and reduces hyperinsulinemia and hyperglycemia [283]. Pancreatic lipid concentrations, particularly of TAGs, are increased in subjects with type 2 diabetes and decreased after bariatric surgery [284]; however, as also suggested for other cells, TAGs are unlikely to be cytotoxic to pancreatic cells. Thus, the ability of β-cells to route fatty acids to TAG synthesis protects against cellular dysfunction and apoptosis [285]. On the other hand, increased FFA influx into β-cells fuels de novo ceramide synthesis, which can impair pancreatic function [163, 286]. This has been identified in the seminal work of the Unger laboratory, where the treatment with either fatty acids (2:1 oleate/palmitate) or C_2_ ceramide increased apoptotic DNA fragmentation in islets isolated from prediabetic rats [161]. This response was abolished upon CerS inhibition by fumonisin B_1_, indicating a critical role of de novo ceramide formation in this process [161]. Accordingly, the lipotoxic effects of fatty acids on β-cells were negated through inhibition of SPT using L-cycloserine in vivo, which ameliorated excessive pancreatic apoptosis and hyperglycemia [32]. It has been proposed that ceramides also account for the profound mitochondrial alterations and ER stress observed in β-cells of the Zucker diabetic fatty rat model, thus promoting pancreatic failure in the pathophysiology of diabetes [287]. Two independent studies demonstrated an additional function of ceramides in cultured pancreatic cells, suggesting a role in modulating glucose-stimulated insulin production [288, 289].

Furthermore, in cultured cells, it was found that chronic exposure of β-cells to supraphysiological levels of glucose and fatty acids promotes the cytotoxic production of selected ceramide species [290, 291]. In INS-1 β-cells, stimulation with glucose and fatty acids induced expression of CerS4 and the formation of specific ceramide species (C_18:0_, C_22:0_, and C_24:1_) concomitant with increased cell death [290]. In turn, inhibition of global ceramide synthesis or selective knockdown of CerS4 in these cells partially prevented palmitate-induced apoptosis [290]. In another study using a mouse insulinoma cell line that exhibits β-cell characteristics, treatment with palmitate increased the expression of CerS5 and CerS6 [291]. Here, the adverse effects of palmitate on β-cells were attributed to both newly generated and salvaged ceramides (C_14:0_, C_16:0_, and C_24:0_), suggesting that these species may be of particular importance in regulating β-cell fate and function also in vivo [291]. However, detailed sphingolipidomic analyses of pancreatic islets in obesity and reports on complementary animal models with pancreas-specific CerS manipulation are lacking. This includes studies investigating the role of ceramides in alternative pancreatic cells, such as α-cells, which are involved in the regulation of systemic glucose metabolism by secreting the peptide hormone glucagon to promote hepatic glucose production, and may also be susceptible to ceramide-induced lipotoxicity in the development of metabolic disease.

### Ceramides in the cardiovascular system

Obesity predisposes to cardiovascular complications such as coronary artery disease, cardiomyopathy, and heart failure, which are primary reasons for the morbidity associated with obesity [292]. Excessive lipid deposition in the vascular endothelium and myocardium in obesity disrupts heart and blood vessel function promoting the development of cardiovascular defects [292]. Emerging studies indicate that plasma concentrations of individual ceramide species bear important prognostic value for cardiometabolic impairments, including atherosclerosis, diabetes, heart failure, and death [293]. Remarkably, specific circulating (dihydro)ceramides in humans were able to predict type 2 diabetes even up to 9 years before disease onset [294] and may also be used for assessment of heart failure and atherosclerosis risk in the general population for primary prevention purposes [295, 296]. More specifically, in the majority of studies, high concentrations of ceramides with C_16:0_, C_18:0_, and C_24:1_ acyl chains and low levels of C_24:0_ ceramides were associated with poor cardiovascular outcomes and increased mortality [297]. On this basis, diagnostic tests to identify subjects at risk of cardiovascular complications by determining particular ceramide ratios and scores are being established [297]. Interestingly, the biomarkers predicting fatal cardiovascular outcomes are proposed to be driven in part by ceramide biosynthesis in hepatocytes [298], adipocytes [299], and intestinal epithelial cells [222].

Ceramides not only can monitor and predict cardiovascular impairment but themselves promote lipotoxic cardiometabolic disease [165]. Accordingly, while ceramides in heart and vasculature are substantially elevated in rodent models of cardiac lipotoxicity and vascular dysfunction, global inhibition of ceramide synthesis improves cardiovascular integrity in obesity [31, 300–302]. A critical role of ceramides appears to be their ability to control blood vessel reactivity through actions in the vascular endothelium [165]. Through actions in endothelial cells lining the vessel intima, ceramides affect vascular tone and contribute to arterial dysfunction. As such, in mouse and bovine coronary arteries ex vivo, treatment with C_2_ ceramide impaired the controlled reduction of vascular tension (vasodilation) [303, 304] and exacerbated blood vessel narrowing (vasoconstriction) in isolated canine cerebral arterial rings [305]. Here, only the treatment with C_16:0_ ceramides, but not C_24:0_ and C_24:1_ ceramides, triggered the constriction of isolated cerebral vascular smooth muscle, indicating ceramide species-specific effects in this process [305]. In addition, increased sphingomyelin hydrolysis has been involved in acute vascular oxygen sensing in the vasoconstrictor response induced by two opposite stimuli, such as hypoxia (in pulmonary and chorioallantoic arteries) and normoxia (in ductus arteriosus) [306]. C_6_ ceramide treatment in cultured human endothelial cells further indicated that ceramides promote oxidative stress by reducing nitric oxide (NO) generation, which is a critical molecule in maintaining basal vascular tone, leading to ROS formation at the expense of NO synthesis [307]. Ceramide-mediated reductions in NO levels are largely due to PP2A-dependent effects on endothelial NO synthase (eNOS) activity, as demonstrated in cultured endothelial cells subjected to high palmitate concentrations [31, 308]. This response can be restored by inhibiting ceramide synthesis using Myriocin or genetic modification of ceramide biosynthetic genes in models of obesity and hyperlipidemia to improve eNOS activity, NO production, and endothelial cell-dependent vasodilation [31, 308, 309].

Furthermore, ceramide accumulation through increased de novo ceramide production may dictate endothelial cell fate and injury [197]. Endothelial cell apoptosis in response to hyperglycemia has been related to the intercellular transfer of high concentrations of C_16:0_ ceramides in large extracellular vesicles derived from nSMase2-dependent sphingomyelin hydrolysis, thereby causing endothelial dysfunction in obesity and diabetes [310]. By correlating C_16:0_ ceramide levels in thoracic adipose tissue and circulation with the deregulation of the vascular redox state and inflammation in human atherosclerotic patients, together with complementary experiments on human tissue ex vivo and primary cultured cells in vitro, it has been suggested that adipose-tissue-derived C_16:0_ ceramides increase the risk of cardiovascular death by acting on endothelial cells to reduce vasodilation, induce inflammation, and promote oxidative stress via eNOS uncoupling [299]. Notably, eNOS uncoupling is increased in patients with endothelial dysfunction resulting from metabolic diseases such as type 2 diabetes mellitus [311], suggestive of a critical role of ceramides in this process.

Endothelial dysfunction in the tunica intima in obese subjects also promotes the development of atherosclerotic lesions in coronary arteries. Here, lipid accumulation in the endothelium and extracellular matrix and inflammatory cell infiltration into subjacent tissue contributes to the onset of atherosclerotic plaque formation. It was recently reported that patients with coronary artery disease show a two-fold increase in TAG content of the right atrial appendage but no alterations in DAGs, associated with a reduction in adipose triglyceride lipase (ATGL) expression, a rate-limiting enzyme in TAG hydrolysis [312]. Additionally, ceramides accumulate in atherosclerotic plaques, implicated in lipoprotein aggregation (in particular LDL) [313]. In human patients, ceramides were enriched in symptomatic versus asymptomatic atherosclerotic carotid plaques, correlating with plaque content of LDL and inflammatory markers [314]. This study also assigned a causal role to ceramides by showing that they promote an inflammatory response in cultured human coronary artery smooth muscle cells, suggesting that ceramides may attract inflammatory cells to the site of atherosclerotic plaque formation [314]. Conversely, inhibition of de novo ceramide synthesis decreased vascular ceramide content and prevented vascular dysfunction and hypertension in high-fat diet-fed mice [31]. Similar beneficial effects were observed upon pharmacologic treatment with Myriocin in the hyperlipidemic and atherosclerosis-prone apolipoprotein E (ApoE)-deficient mouse model, which prevented the development of atherosclerotic plaques and enabled the regression of established lesions [315]. Interestingly, circulating ceramides derived from the intestine promote the development of atherosclerosis, and decreasing plasma ceramides through suppression of the intestinal FXR/Smpd3 axis reduced lesion areas in the aortas of ApoE-deficient mice [222]. In turn, replenishment of C_16:0_ ceramides could partially reverse these improvements, suggesting a specific role of C_16:0_ ceramides in atherosclerosis [222]. Collectively, it is emphasized that lowering plasma ceramide levels may be an effective strategy to improve vascular health in obesity. Still, causal relationships between specific ceramide molecular species and atherosclerosis need to be worked out.

Besides the critical role in the vasculature, ceramides are necessary for maintaining cardiac integrity, as exemplified by the diminished heart function in mice with heart-specific *Sptlc2* deficiency [316]. Similar to ceramide depletion, also the accumulation of ceramides in the heart is associated with cardiotoxicity, and ceramide content increases during obesity-related progressive cardiac remodeling and dysfunction [317, 318]. It has been demonstrated that diets rich in myristate promote early development of cardiac hypertrophy, left ventricular systolic and diastolic dysfunction, and autophagy due to increased CerS5-dependent C_14:0_ ceramide synthesis [319]. Accordingly, in isolated primary cardiomyocytes, myristate but not palmitate could induce CerS5-dependent hypertrophy and autophagic flux, indicating that CerS5-derived C_14:0_ ceramides may be involved in cardiomyocyte autophagy and lipotoxic diabetic cardiomyopathy [319]. In a follow-up study, a longer exposure to the diet increased myocardial C_18:0_ and C_18:1_ ceramides but also CerS2-derived C_22:0_ and C_24:0_ ceramide species [320]. Interestingly, while the overexpression of either CerS2 or CerS5 triggered cerdiomyocyte apoptosis, only the overexpression of CerS2 induced mitochondrial dysfunction and mitophagy in cardiomyocytes; however, it did not affect hypertrophy, suggesting that CerS2 and CerS5 have distinct roles in this process [320]. 

Myriocin treatment and the selective reduction of specific ceramides (C_16:0_, C_24:0_, and C_24:1_) in the heart of mice with ischemic cardiomyopathy reduced ventricular remodeling, fibrosis, and macrophage content following myocardial infarction [318]. In another study, Myriocin treatment significantly blunted the increase of myocardial ceramides in the lipid-overloaded heart of a mouse model of dilated cardiomyopathy, i.e., in mice expressing glycosylphosphatidylinositol (GPI)-anchored human lipoprotein lipase (LpL^GPI^) [80]. The reduction of ceramide de novo synthesis in these animals improved myocardial glucose oxidation rates, cardiac efficiency, and survival and reduced the expression of heart failure markers [80, 321]. Similar beneficial effects were obtained after Myriocin treatment in diet-induced obese mice, which decreased myocardial ceramide content and improved glycolysis and glucose oxidation in isolated aerobic perfused working hearts in the presence of insulin [302]. From a mechanistic point of view, myocardial ceramides trigger ER stress and apoptosis, decrease mitochondrial function, and promote insulin resistance, thereby contributing to the pathophysiology of cardiomyopathy [322–324]. Collectively, it is predicted that targeting the production of specific ceramide species may have profound beneficial cardiovascular effects to improve obesity-associated cardiometabolic complications.

### Ceramides in hypothalamic neurons

In the central nervous system, a dynamic network of neurons located in spatially distinct areas of the hypothalamus responds to hormonal and nutritional cues in order to compute the organism’s energy state and adapt food intake and metabolic rate [325]. Different hypothalamic nuclei, including the arcuate nucleus (ARC), the ventromedial nucleus (VMH), and the lateral hypothalamic area (LHA), have profound roles in regulating food intake, glucose homeostasis, and metabolism [326]. Acute high-fat diet feeding and chronic over-nutrition are associated with a rise in hypothalamic lipid concentrations that promotes a decline in sensitivity toward hormonal input within the hypothalamic melanocortin circuitry [327, 328]. Interestingly, central actions of both the adipokine leptin and the gastric hormone ghrelin involve changes in hypothalamic ceramide content to pursue their respective effects on food intake, indicating critical regulatory functions of ceramides in this process [329, 330].

Given the highly heterogeneous composition of neurons in the hypothalamus, it is likely that significant differences in ceramide species occur across different neuronal populations, which has not been resolved thus far. Still, evidence now clearly indicates that ceramides mediate many of the adverse effects of fatty acids on neuronal integrity, contributing to metabolic impairment when they accumulate in the hypothalamus, as is the case in hyperlipidemic and high-fat diet-fed animal models [331]. Studies revealed increases in selected ceramide species in the hypothalamus of high-fat diet-induced obese mice (C_18:0_, C_22:0_, C_24:0_), diabetic and dyslipidemic rats (C_16:0_, C_18:0_, C_20:0_), and in the lipid-overloaded hypothalamus of obese Zucker rats (C_16:0_, C_18:0_) [135, 332, 333]. Intriguingly, hypothalamic accumulation of ceramides appears to be sexually dimorphic, as elevated levels of ceramides were only found in the hypothalamus of males compared to female mice following consumption of high-fat diets [334]. Here, ceramide levels correlated with reduced PGC-1α and estrogen receptor α (ERα) to promote hypothalamic inflammation and myocardial dysfunction in a sex-specific manner [334]. Sex dimorphisms are primarily due to hormonal differences between sexes, e.g., ovarian steroids that deeply affect metabolic networks in females [334]. Accordingly, estradiol (E2) controls ceramide content in the hypothalamus of female rats [335]. E2 is implicated in sexual maturation and regulates food intake through effects on proopiomelanocortin (POMC)-expressing neurons in the ARC as well as BAT thermogenesis through impact on the VMH [336]. Ovarian insufficiency, in turn, is associated with ceramide accumulation in the mediobasal hypothalamus of rats and hyperphagia, reduced energy expenditure, and increased weight gain [335]. Conversely, central E2 treatment reduces hypothalamic ceramide content, possibly via AMPK, and ameliorates ceramide-induced lipotoxicity and ER stress by affecting the sympathetic nervous system and BAT thermogenesis [335]. Ceramide accumulation in the hypothalamus was also observed in mice with deficiency for the lipoprotein lipase (LPL) in astrocytes, which represent important sites of brain lipid sensing [337]. Astrocytic ceramide accrual increased hypothalamic immunoreactivity of the appetite-regulating agouti-related peptide (AgRP) and ER stress marker gene expression in conjunction with elevations in food intake, body weight gain, adiposity, and glucose intolerance [337]. Furthermore, transcript levels of sphingolipid-metabolic genes, including that of specific *CerSs*, are increased in particular neurons of diet-induced obese mice, as was recently suggested from transcriptomic analysis in POMC neurons [338].

Ceramides derived from endogenous ceramide synthesis elicit direct effects on hypothalamic neurons. In cultured hypothalamic GT1-7 neuronal cells, palmitate-dependent increases in ceramide content decreased insulin sensitivity [339]. Myriocin treatment or *Sptlc2* knockdown in these cells abolished the inhibition of insulin sensitivity, indicating that ceramides produced through de novo synthesis have critical roles in the manifestation of neuronal insulin resistance during fatty acid excess [339]. Accordingly, ICV infusion of C_2_ ceramides in obese Zucker rats impaired and Myriocin infusion improved hypothalamic insulin sensitivity and systemic glucose tolerance, which was attributed to increased glucose-stimulated insulin secretion and β-cell mass [339]. TLR4 signaling was found essential for lipid-induced hypothalamic ceramide accumulation, and TLR4 deficiency in mice prevents hypothalamic ceramide accrual in response to lard oil infusion, protecting the animals from fatty acid-induced insulin resistance and systemic glucose intolerance [192, 340]. Consistently, inhibition of IKK-β in obese animals can lower hypothalamic ceramide concentrations, leading to similar beneficial metabolic outcomes, demonstrating the functional relevance of inflammatory signals in regulating hypothalamic ceramide turnover and metabolic homeostasis.

Campana et al. found a positive impact of PKC inhibition on fatty acid- and ceramide-induced insulin resistance in cultured hypothalamic neurons [339]. Here, pharmacological inhibition of PKCs or expression of a dominant-negative version of PKCζ counteracted the inhibition of AKT phosphorylation induced by either C_2_ ceramide or palmitate treatment in GT1-7 cells [339]. Ceramide-mediated PKCζ regulation may thus be central to modulation of insulin sensitivity also in hypothalamic neurons, but this has not been confirmed in vivo. The detrimental effects of ceramide accrual in the hypothalamus are also partly mediated through ER stress and associated inflammation. In mHypoE-N42 cells, inhibition of de novo ceramide formation through L-cycloserine treatment reduced palmitate-induced inflammation [341]. Other fatty acids, namely oleic- and eicosatetraenoic acid, showed anti-inflammatory effects by decreasing palmitate-induced ceramide build-up [341].

Contreras et al. found that hypothalamic ceramides promote ER stress in the VMH, thereby reducing sympathetic tone that impairs BAT metabolic function in the control of systemic energy metabolism [135]. In this study, ICV treatment of rats with C_6_ ceramide increased C_16:0_ ceramide content in the mediobasal hypothalamus, which was associated with increased expression of inflammatory markers and elevated ER stress [135]. Central administration of C_6_ ceramide reduced sympathetic nerve activity, diminished the thermogenic capacity of BAT, and impaired systemic insulin sensitivity [135]. Similarly, increasing ceramide synthesis by overexpression of SPTLC1/2 in the VMH increased ER stress in hyperthyroid rats [210]. Downregulation of SPTLC1 in VMH, in turn, ameliorated ER stress and improved metabolic health in ovariectomized rats [335]. Decreases in hypothalamic ceramide content induced by T3 treatment were associated with reduced hypothalamic ER stress, in conjunction with improved BAT mitochondrial activity, thermogenesis, and metabolic homeostasis [210]. Together, these studies indicate that ceramide accumulation in the hypothalamus, as present in obesity, promotes hypothalamic ER stress, particularly in the VMH, leading to systemic metabolic impairments.

In addition, a role of ceramides in the PVH concerning sexual maturation in obesity has been identified [342]. In contrast to undernutrition that delays puberty, childhood obesity often accelerates puberty onset, linked to a higher disease burden later in life [343]. It was recently found that de novo synthesis of nearly all ceramide species is increased in the PVH of early-onset obese female rats, leading to ceramide accumulation and precocious puberty [342]. Central administration of C_6_ ceramides induced pubertal precocity, while it was delayed in lean female rats after inhibiting ceramide synthesis using Myriocin [342]. In particular, the PVH has been proposed as a critical hypothalamic region for transmitting sympathetic neural information to the ovary to control ovarian maturation and function [342, 344]. Accordingly, PVH de novo synthesized ceramides triggered an increase in ovarian sympathetic tone in early-overfed rats through interplay with kisspeptin in a non-canonical pathway of the central control of puberty [342].

Collectively, there is emerging evidence for a specific role of hypothalamic ceramides in obesity-related deregulations. However, the particular neuronal populations and potential other cell types in the hypothalamus in which specific ceramides affect metabolic homeostasis have not yet been clearly defined.

## Is it possible to modulate ceramide metabolism for the treatment of obesity-related diseases?

As highlighted in this article, plasma and tissue ceramide levels increase during obesity development associated with the onset and progression of metabolic diseases in both animal models and humans. An obvious question arising from these findings is whether inhibiting ceramide synthesis or stimulating ceramide degradation would provide strategies to efficiently improve metabolic health and treat obesity-related disorders in human patients. Results from rodent studies support this idea, showing that the disruption of ceramide synthesis protects from insulin resistance and other metabolic complications of obesity and can reverse these pathologies when achieved in an inducible manner [12, 29, 30, 33]. The fact that conditional, tissue-specific reduction in ceramide content in obese rodent models is sufficient to improve metabolic homeostasis emphasizes the possibility of developing tissue-restrictive drugs for selective inhibition of ceramide production. Thus, peripherally acting pharmacotherapeutics may circumvent adverse reactions associated with ceramide-lowering interventions such as neurodegenerative processes in the brain [345]. However, inhibitors to modulate components of the ceramide metabolic pathway in humans with tolerable side effects are not yet available. Therefore, to the authors’ knowledge, there is currently no publicly available information about clinical experience with specific inhibitors of ceramide biosynthetic enzymes in obese and diabetic patients.

Strategies to improve metabolic health, such as the treatment with insulin-sensitizing agents (metformin and pioglitazone), acute exercise, or weight loss, reduce ceramide levels in tissues and circulation [275, 346, 347]. In addition, it was found that people consuming “healthier diets,” such as Nordic or Mediterranean diets, exhibit lower circulating ceramide levels and a lower risk for cardiovascular disease and diabetes as compared with those consuming more typical foods [348, 349]. Plasma ceramide levels are also reduced following the application of statin-based pharmacotherapies in patients with metabolic syndrome and coronary artery disease, used to treat hypercholesterolemia for primary and secondary prevention of cardiovascular disease [350, 351]. Gastric bypass surgery in obese subjects also lowers circulating ceramide levels [352], and this intervention is applied for the prevention and remission of diabetes, hypertension, and dyslipidemia [353]. Collectively, although the studies only show correlations between ceramides and metabolic integrity, it is interesting to speculate that these interventions cause beneficial metabolic outcomes by affecting ceramide content. In addition, they show that ceramide levels in humans are indeed modifiable, highlighting the potential of ceramide-lowering interventions in future clinical settings. It is also interesting to note that extracts from the endoparasitic fungus *Cordyceps sinclairii*, from which the SPT inhibitor Myriocin was initially isolated, are commonly applied to patients in traditional Chinese medicine to treat an array of health impairments, including diabetes [354]. However, long-term treatment with Myriocin itself can exert adverse health effects, e.g., hepatoxicity, as shown in male Wistar rats [355]. The need to identify novel and more specific inhibitors of ceramide synthesis is thus clearly emphasized.

It should be noted that the complete inhibition of global ceramide formation poses considerable risk of adverse side effects due to the multifaceted cellular functions of ceramides and their sphingolipid derivatives. This is also exemplified by the embryonic lethality in mice with homozygous deletion of *Sptlc1* and *Sptlc2* [356], the development of liver cancer upon hepatocyte-specific knockout of *Sptlc2*, and the development of inflammatory bowel disease and early lethality when *Sptlc2* is deleted in an inducible and intestine-specific manner [357, 358]. Along the same lines, mice with homozygous deletion of *Degs1* reveal an incompletely penetrant lethality, and the surviving animals show growth retardation with several health complications [12]. Similarly, *DEGS1* missense mutations in humans cause severe neurological disorders [359–361]. Nevertheless, the development of pharmacological approaches to partially inhibit DES1 in obesity to treat cardiometabolic diseases is being pushed forward [362]. A similar suggestion has been made for CERS6 to avoid the broad spectrum of possible side effects [35]. Indeed, the CerS enzymes could offer attractive drug targets for obesity and diabetes therapy [363]. This suggestion is mainly based on the observation that human CERSs and the regulation of the corresponding ceramide products during obesity development are conserved from mice to humans and that the inhibition of specific CerSs is sufficient for alleviating obesity-associated metabolic dysfunction in related murine studies [363]. Specifically, the disruption of C_16:0_ ceramide synthesis either by the inducible deletion of *CerS6* or treatment with *CerS6*-specific ASOs can improve systemic glucose metabolism in obese and insulin-resistant mice [26, 243]. Similarly, pharmacological inhibition of CerS1 in obese mice using P053 treatment to reduce C_18:0_ ceramide content predominantly in skeletal muscle efficiently improves lipid metabolism [34], and no adverse effects as a consequence of either intervention have been reported to date.

When CerS enzymes are selected as targets for pharmacological intervention, it is important to consider the different physiological roles of their ceramide products and the consequences of reducing them, which may result in a compensatory increase in other cytotoxic ceramide molecular species. In this context, it is also important to reiterate that even ceramides with a specific acyl chain length can have different physiological functions and pathological effects depending on their intracellular localization, which is partly determined by the specific synthesizing CerS [26]. This specificity could provide a highly advantageous opportunity to target only restricted pools of ceramide species that exert adverse effects on metabolic homeostasis while leaving other populations unaffected and available to maintain critical cellular processes. The development of such specific inhibitors of ceramide synthesis could pave the way for sophisticated novel therapeutic strategies to combat the epidemic of obesity and its comorbidities.

## Conclusion and future perspectives

Ceramides are bioactive lipids that exert a plethora of metabolic functions through their unique biophysical properties in membranes and their abilities to control intracellular signaling pathways in part through binding to regulatory proteins. As discussed herein, the biological roles and pathological effects of ceramides highly depend on the molecular composition of the ceramide species, partly defined by the length of the acyl chain. Recent advances in high-resolution mass spectrometry-based lipidomics have laid the foundations for dissecting the complexity of the acyl chain ceramide distribution in membranes and its dynamic regulation upon varying physiological conditions. However, we still face limitations in the technical abilities to study ceramides in small cell populations and at the single-cell level, which will be crucial to understand the cell-type-specific regulation of ceramides in greater detail in vivo. The need for a cell to exhibit this striking diversity is not fully understood, as are the stimuli that control membrane ceramide plasticity. Still, it underscores the need for multilayered lipid-regulated mechanisms to fine-tune biological processes through appropriate alterations in membrane dynamics and membrane-emanating signaling cascades in response to a wide array of stimuli. In this light, ceramides may act as critical metabolic messengers to control lipid and glucose homeostasis, mitochondrial plasticity, and inflammatory signaling in conditions of fatty acid excess. We have discussed studies indicating that several metabolism-regulatory pathways (e.g., adiponectin receptor signaling) mediate their cellular actions by modulating ceramide turnover and that deregulation within these processes impacts metabolic health depending on specific ceramide species. In accordance, it has been consistently demonstrated that ceramide metabolism is altered in obesity and that the accumulation of selected ceramide molecular species can have detrimental pathological consequences in a tissue-specific manner.

Whereas initially, only total amounts of ceramides in cell and tissue homogenates were reported, and interventions were aimed at inhibiting general ceramide synthesis, studies over the past decade have demonstrated that quantifying total ceramide levels is insufficient to identify alterations in ceramide metabolism; this is due to the distinction of specific acyl chain length molecular species that can be regulated independently of each other and exert defined metabolic and organ-specific roles. In particular, CerS6-derived C_16:0_ ceramides in the liver and BAT and CerS1-derived C_18:0_ ceramides in skeletal muscle evolved as critical regulators of metabolic integrity and dysfunction in obesity. Defining the roles of the relevant ceramide species also in other tissues, such as the pancreas, heart, vasculature, and hypothalamus, will provide additional insights into the tissue-specific functional versatility of ceramides, allowing us to unravel further the immense complexity of lipid-based mechanism in maintaining or disturbing metabolic homeostasis in obesity. Characterizing cell-type-specific CerS knockout mouse models and developing CerS-selective inhibitors to modulate the synthesis of specific ceramide molecular species will be important in the future. Nevertheless, recent observations indicate that—depending on the research objective—simply distinguishing between levels of particular ceramides is no longer sufficient, given that the same molecular species can be regulated differently in different cellular compartments. The sub-compartmentalization of these ceramides can have specific biological roles with independent pathological consequences when they accrue, further supporting the need for analyses of ceramides within their relevant subcellular locations. In addition, although the physicochemical properties of ceramides have been subject to extensive research in vitro and studies on the cellular actions and pathological properties of ceramides have emerged, definite causal relationships often remain to be established. By linking both research areas in the future, it is expected that new avenues can be explored to dissect the biological functions of particular acyl chain ceramide species in obesity.

This article addresses the impact of ceramides with specific acyl chain lengths in obesity. However, it has to be kept in mind that further layers of ceramide complexity confer additional functional specificity in physiology and disease. This involves (a) varying sphingoid bases, (b) acyl chain unsaturation, and (c) ceramide headgroup modification (e.g., phosphorylation and glycosylation). Moreover, it should by no means be ruled out that other lipid species make a decisive contribution to metabolic regulation and promote lipotoxicity in the deterioration of metabolic homeostasis, such as already proposed for sn-1,2-DAGs [364]. Nevertheless, as hopefully appreciated by the reader, the research on ceramides in obesity and their specific roles in metabolic disease pathogenesis has begun to evolve. Further research is undoubtedly required to unravel the precise mechanisms by which specific bioactive ceramide species are regulated in obesity, and to better understand how they modulate metabolic processes in the tissue-specific (de)regulation of metabolic homeostasis.

In particular, as the obesity epidemic continues to spread and cases of obesity-related metabolic diseases inexorably increase, new targets must be found for therapeutic intervention. Restraining acyl chain length-specific ceramides in production or action could be a novel approach in this context. The identification of the specific ceramide target proteins in distinct cellular compartments and the prevention of ceramide-(binding)-induced deregulations in downstream signaling pathways might bring us closer to achieving this goal. Furthermore, pharmacological inhibition of specific CerS enzymes in obesity—promising candidates are CerS1 and CerS6—holds the potential to treat obesity-related metabolic disease while circumventing the adverse consequences associated with inhibiting global ceramide production.

## Box 1

Box 1: ceramide synthases in a nutshell Six mammalian ceramide synthase enzymes (CerS1-6) produce dihydroceramides of specific acyl chain lengths (C_14_-C_26_) in the de novo or salvage pathway of sphingolipid formation, defining a large proportion of the ceramide structural and functional heterogeneity [21]. The identification of CerSs dates back more than 25 years to the cloning of the longevity-assurance gene (*lag1*), which was named according to its role in regulating lifespan in *S. cerevisiae* [365], and encodes an enzyme that promotes ceramide synthesis much like its close cognate Lac1 [366]. A single gene called *Schlank* encodes a protein with ceramide synthase activity in *Drosophila*, whereas the *C. elegans* genome comprises three distinct ceramide synthase genes, namely *hyl-1*, *hyl-2*, and *lagr-1* [367]. Deletion of *hyl-1* and *hyl-2* produces substantially different phenotypes in the anoxia response, demonstrating the exclusive functions of their ceramide products depending on the length of the acyl chains in *C. elegans* [368]. In mammals, six different mammalian longevity-assurance genes (*Lass1-6*) were described and later renamed ceramide synthases (*CerS1-6*) according to the biochemical roles of their encoded proteins [369]. The substrate specificity of the CerS enzymes toward certain fatty acyl-CoAs is dictated by a defined sequence of eleven amino acids in the C-terminal portion of the functional TRAM-Lag1-CLN8 (TLC) domain [370]. CerSs show distinct tissue-expression patterns partly underlying the tissue-specific distribution of the corresponding (dihydro)ceramide species [371]. It has been found that *CerS1* mRNA is most prominently expressed in brain and muscle tissue, concomitant with high relative amounts of its specific ceramide product (C_18_ ceramide) in brain and muscle extracts [28]. CerS2 shows substrate preference for C_22_ and C_24_ acyl-CoA and is ubiquitously expressed, with the highest mRNA levels in the liver and kidney [371]. CerS3 expression is restricted to testis and skin and required to form ultra-long-chain ceramides, including the chain length C_26_ [371, 372]. CerS4 shows substrate preference for C_18_-C_20_ acyl-CoA and is highest expressed in heart, leukocytes, liver, and skin [371]. CerS5 and CerS6 are expressed in a variety of different organs and share an overlapping specificity for the generation of C_14_ and C_16_ ceramide species [371, 373, 374]. However, ceramide levels do not always correlate with CerS abundance, indicating a much more complex regulation of CerS activity, including protein–protein interactions, phosphorylation, glycosylation, acetylation, and allosteric modulation by sphingolipid-binding [375]. For example, the formation of CerS homo- and heterodimers via a defined C-terminal motif is involved in the mutual regulation of CerS activity [376]. Unfortunately, post-translational modifications that dictate altered CerS activity in obesity are barely investigated. While the role of CerSs in ceramide synthesis has been a significant focus, a homeodomain was identified in CerS2-CerS5, which in the *Drosophila* CerS-homologue Schlank confers transcription-regulatory properties to regulate lipid homeostasis independent of ceramide synthesis [377, 378]. A similar role for mammalian CerSs has been suggested but awaits experimental validation [377, 378].

## Box 2

Box 2: what we need to consider when studying ceramide(s) (synthases) in vivo Research on the exclusive roles of different ceramide species often utilizes genetic or pharmacological approaches to modulate individual CerSs responsible for the production of ceramides with specific acyl chain lengths. Such studies have demonstrated the functional complexity of the mammalian CerS enzyme family and their respective ceramide products, with different knockout or overexpression models displaying markedly different phenotypes [16]. However, in experiments targeting gain- and loss-of-function of ceramide biosynthetic genes or enzymes, secondary changes in the levels of non-specific sphingolipid products may occur and must be considered in relation to the observed phenotypes. For example, CerS2 deficiency in mice leads to a decrease in the levels of C_22:0_-C_24:0_ ceramides and a compensatory increase in C_16:0_ ceramide synthesis, with the latter accounting for a significant portion of the metabolic consequences of CerS2 deficiency [70, 71, 93]. It is undoubtedly important to analyze the entire spectrum of ceramides before specific conclusions are drawn about a particular ceramide molecular species. In addition, the accumulation of sphingoid long-chain bases as a result of CerS deficiency may exert cytotoxicity independent of changes in the corresponding ceramide products [379]. Thus, mice carrying a CerS1 loss-of-function mutation develop early-onset cerebellar ataxia and Purkinje cell degeneration, supposedly due to sphinganine accumulation rather than reduced C_18:0_ ceramide levels in the brain [379]. Similarly, changes in ceramide content are assumed to result in the alterations of other downstream sphingolipid species that may affect cellular metabolism. Detailed analysis of the cellular (sphingo)lipidome, wherever feasible, would help gather information on secondarily altered ceramide derivates and other lipid metabolites of potential relevance. Furthermore, when comparing ceramide profiles and the corresponding physiological results across different studies, it is important to consider the influence of environmental factors on the ceramide content in the model organism under investigation. For example, dietary composition and the microbiota strongly affect endogenous ceramide turnover rates. Thus, differences in animal husbandry and dietary fat content alone may result in significant differences in tissue ceramide profiles, translating into different phenotypic expressions.

## Data Availability

No data sets were generated for this review.
